# Comparative phenotyping of surface markers and glycans in murine and human platelet-derived extracellular vesicles

**DOI:** 10.1016/j.rpth.2026.103414

**Published:** 2026-03-20

**Authors:** Olga An, Friedrich Reusswig, Viola Krenzlin, Carsten Deppermann, Dianne E. van der Wal

**Affiliations:** 1 Center for Thrombosis and Hemostasis, University Medical Center of the Johannes Gutenberg University Mainz, Mainz, Germany; 2 Research Center for Immunotherapy, University Medical Center of the Johannes Gutenberg University Mainz, Mainz, Germany; 3 ANZAC Research Institute, Concord Repatriation Hospital, Concord, NSW, Australia; 4 School of Medical Sciences, Faculty of Medicine and Health, University of Sydney, Sydney, Australia

**Keywords:** disease models, animal, extracellular vesicles, glycoproteins, humans, mice, blood platelets, glycosylation, glycomics, lectins

## Abstract

**Background:**

Platelet-derived extracellular vesicles (PEVs, 100-1000 nm in size) are released from activated platelets. They are important carriers of signaling molecules, lipids, and proteins. They interact with immune and tumor cells and, thus, hold great promise as drug delivery tools. They express a plethora of unique surface glycans, and the majority of PEVs in circulation are nonprocoagulant and phosphatidylserine-negative. Despite an increasing number of studies on PEVs, receptor and glycosylation profiles of murine and human PEVs have not been characterized systematically.

**Objectives:**

To characterize large PEVs (l-PEVs) generated from human and murine platelets using double stimulation of glycoprotein VI (GPVI) and PAR1/4 pathways, as well as ionophore A23187.

**Methods:**

Surface glycoprotein and glycosylation profiles of human and mouse l-PEVs were determined using flow cytometry, western blotting, and lectin array.

**Results:**

Interestingly, murine samples showed a significantly altered glycoprotein expression and glycosylation compared with human l-PEVs. Following stimulation, GPVI, CD41/CD61, and CD62P were expressed to a lower extent on murine l-PEVs. CD42b (GPIb) was not highly abundant on l-PEVs from both species. The pan-inhibition of metalloproteinases resulted in recovery of CD42b but not GPVI on l-PEVs. Moreover, the analysis of surface and the total content of carbohydrates of the l-PEVs showed species-specific surface glycan exposure. We also detected only minor differences in phenotypes of l-PEVs generated from buffy coat or fresh blood and between l-PEVs generated from blood with different types of anticoagulation.

**Conclusion:**

We identified specific differences in the phenotype of mouse vs human l-PEVs, which contribute to our understanding on the role of PEVs, which should be considered when designing future PEV studies, for example, in commonly used murine models of disease.

## Essentials

• Platelet-derived extracellular vesicles carry molecules to interact with immune and tumor cells.
• Using flow cytometry, we analyzed receptors and glycosylation of large PEVs of mice and humans.
• Expression of main platelet glycoproteins and carbohydrates were different between species.
• Our findings should be considered when designing extracellular vesicle studies.

## Graphical abstract

**Figure:**
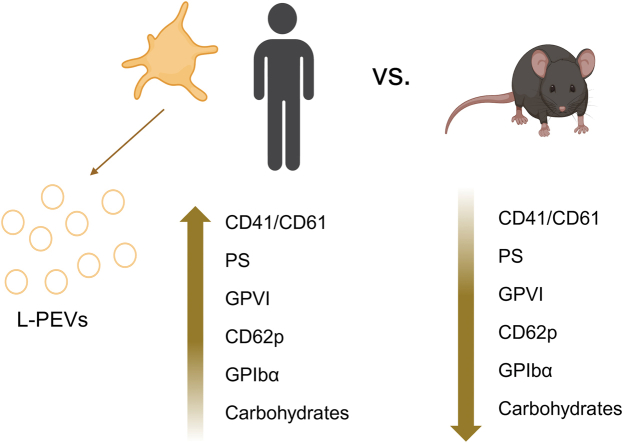

## 1 Introduction

Upon activation, platelets release extracellular vesicles (EVs) in the range of 100 to 1000 nm [1]. Platelet-derived extracellular vesicles (PEVs) carry different RNA species, enzymes [2], proteins, and other biologically active substances that can influence immune cells [3]. They contribute not only to thrombus formation but also to bone marrow remodeling [4], tumor progression [5], (chronic) inflammation, and thromboinflammation [6].

A variety of PEV surface molecules have been described [7], including the main platelet glycoproteins (GPs), α_IIb_β_3_ integrin (CD41/CD61), CD42b (GPIb), and GPVI [7]. P-selectin (CD62P) can be present on the EV surface when released from activated platelets [8]. In circulation, only a minority of (CD41^+^) PEVs are procoagulant and expose phosphatidylserine (PS) [9,10]. Nevertheless, PS-positive PEVs play a significant role in the amplification of the coagulation response due to their higher procoagulant potential compared with platelets [11]. PS on EVs not only allows the binding of coagulation factors but is also an important “eat-me” signal for PS-counter receptors on phagocytes [12], such as TIM-4, lactadherin [13], and CD36 [14].

Platelet clearance is tightly connected to their surface glycans [15,16]. Young platelets are covered with sialic acid [17], which they lose during their short lifespan. Upon loss of sialic acids (desialylation), underlying residues from either *O*- (simple) or more complex *N*-linked glycans, for example galactose residues, become exposed [18]. Hepatocytes and macrophages (eg, Kupffer cells in the liver) recognize β-galactose and *N*-acetylglucosamine (GlcNAc) via their Ashwell–Morell, Mac-1 (CD11b/CD18; α_2_β_M_), and macrophage galactose lectin (CD301) receptors [16,19]. PEVs are also highly glycosylated [20,21] and are modified during storage of platelet concentrates [21]; specifically, PS^+^ PEVs lose α-2,3-linked sialic acid during storage, and desialylation is linked to a more procoagulant PEV phenotype [20]. Furthermore, surface glycan modifications influence biodistribution of EVs *in vivo* [22].

The expression of the main surface receptors [7], as well as some of the content [23,24] of PEVs, has been described in patients and healthy donors. The expression of membrane GPs, including CD42b, CD62P (P-selectin), PS, and integrin α_IIb_β_3_, has been demonstrated by previous studies [25,26]. However, a full analysis of mouse PEVs and an in-depth comparison of human- and murine-derived PEVs has not been performed to date. It was shown that thrombin stimulation led to the generation of large PEVs (l-PEVs) [8,23], which are enriched in CD41. Moreover, comparison of PEVs generated by the calcium ionophore ionomycin and a combination of thrombin receptor and GPVI agonists revealed differences in the protein content of respective particles [23], including myosin light polypeptide 6 and myosin regulatory light chain 12A. Ionophore stimulation generated more vesicles with higher protein content. Interestingly, the protein cargo was not different between PEVs generated following stimulation with different agonists [23]. These findings highlight the heterogeneity of PEV populations that show distinct interactions with immune cells.

When focusing on platelets, important differences between murine and human platelets have been noted previously. Murine platelets are significantly smaller in diameter, they have less granules, their lifespan is shorter, and some aspects of the signaling machinery differ, for example, the SRC kinase and P2Y12 receptor show differential expression [[27], [28], [29]]. Murine platelets show increased expression of GPVI [30]; however, they lack FcγRIIA and the thrombin receptor PAR1 [29]. Nevertheless, human and murine platelets share a lot of similarities in the signaling pathways and the expression of main platelet receptors (CD42b and integrin α_IIb_β_3_). This is why murine models are widely used in translational studies to decipher mechanisms of cardiovascular diseases involving platelets.

Increased PEV numbers are observed in a plethora of diseases where PEVs of different phenotypes contribute to development of coagulopathy [31], recruitment of immune cells [32], and tissue regeneration [2]. Severe coagulopathy in trauma is associated with the release of procoagulant (PS^+^) PEVs with high thrombin-generating potential [31]. Fusion of CD42b^+^ PEVs with monocytes promotes their attraction and firm adhesion with damaged endothelium [32]. Protein and RNA cargo include various growth factors [24], contributing to tissue regeneration [33].

Besides studying the role of PEVs in (patho)physiology, biodistribution studies of EVs are of high interest for deciphering the therapeutic potential of EV preparations [34,35]. EVs derived from tumor cells are used in studies of biodistribution of vesicles [33,36], which show that the main sites of accumulation are the liver, spleen, lung, and tumor tissue [36]. Moreover, since PEVs preferentially bind to tumor cells, they have been studied as delivery tools of anticancer drugs [37]. Study of biodistribution of labeled murine PEVs revealed their accumulation in lymphoid organs of recipient mice. PEVs are also found to bind to immune cells in peripheral blood [38]. Nevertheless, much interest remains in the distribution of human EVs, and several studies involve administration of human nano (drug) medicine into mice models. Humanized murine models are also widely used in studies that aim at improving blood products for transfusion, including platelet concentrates containing large numbers of PEVs [39]. Besides humanized mouse strains, immunocompetent mice have been used in biodistribution studies [36], which leaves the question whether cell–PEV interactions would be comparable between humans and mice.

Considering aforementioned interspecies differences with regard to platelets and the immune system [40], it is important to understand the different PEV phenotypes and their implications for the biodistribution of human vs murine PEVs. The heterogeneity in the abundance of surface adhesion and/or (glyco)proteins might affect cellular interactions with (immune) blood cells. Moreover, current biodistribution studies of small or large EVs use fluorescent or isotope labeling of EV membranes [33,36] and do not focus on the surface markers responsible for recognition of those particles by other cells. Therefore, in this study, we aimed to compare the phenotype of human and murine PEVs.

## 2 Methods

### 2.1 Materials

BD Trucount tubes (BD Biosciences) were used for EV enumeration. Antibodies against human platelet GPs were from BioLegend (CD61-FITC, Annexin V-FITC, CD42b-PE, and CD62P-PE) or Invitrogen (GPVI-eFluor660). Anti-murine antibodies were from BioLegend (CD41-AlexaFluor647) or Emfret (CD42b-DyLight649, CD62P-FITC, and GPVI-FITC). *Ricinus communis* agglutinin 1, succinylated wheat germ agglutinin, *Sambucus nigra*, *Maackia amurensis*, concanavalin A, and *Ulex europaeus* agglutinin (all fluorescein-conjugated) were from Vector Laboratories. Isotype controls along with unstained controls were used for antibody or lectin-positive populations.

### 2.2 Platelet collection

Human platelets were isolated from buffy coats (BCs) obtained from fresh whole blood collections from the Transfusion Center of the University Medical Center Mainz (ethics committee of the state medical association of Rhineland-Palatinate vote number 2024-17880). Sequential centrifugation in presence of 3.8% sodium citrate was used to isolate platelets (100*g* for 8 minutes; twice 400*g* for 5 minutes, without brake). Murine blood was obtained from 10- to 12-week-old C57BL/6 mice. Retro-orbital bleeding was performed into heparin-containing tubes (in accordance with approval number G24-1-079 of the Landesuntersuchungsamt Rheinland-Palatinate). Platelets were isolated using centrifugations (twice 60*g* for 6 minutes; 650*g* for 5 minutes, without brake) in the presence of 10 U/mL of heparin (Leo Pharma), 1 U/mL of apyrase (Sigma Aldrich), and 0.5 μM of PGI_2_ (Cayman Chemical) [41]. Platelet count was determined using a Sysmex KX-21N hematology analyzer. Platelets were diluted to 200 × 10^6^/mL (human and murine) in Tyrode’s buffer (140 mM NaCl, 0.5 mM NaHCO_3_, 3 mM KCl, 0.5 mM MgCl_2_, 10 mM d-glucose, 10 mM HEPES, pH 7.35) with 2 mM of CaCl_2_ filtered with 0.22-μm PES sterile filter (Carl Roth GmbH).

### 2.3 Generation of PEVs

**Figure 1:**
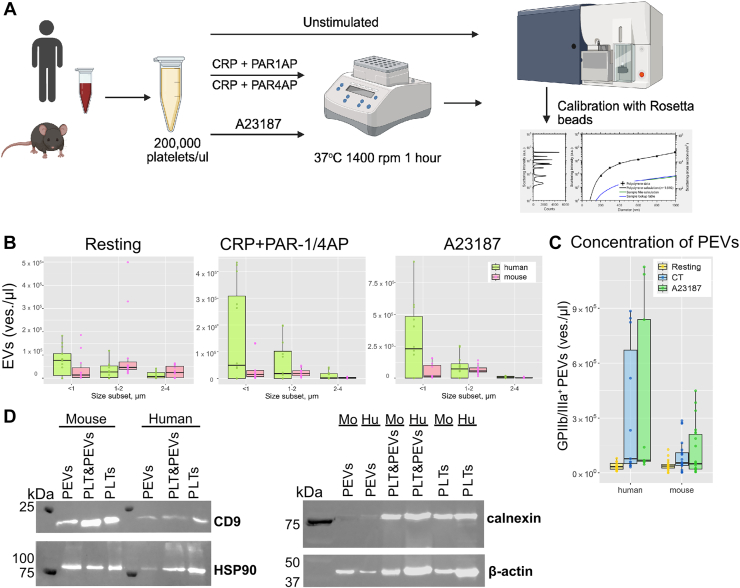
Characteristics of human and murine platelet-derived extracellular vesicles (PEVs). (A) Large PEVs (l-PEVs) and platelets from unstimulated and activated (CT, A23187) samples of mice and humans were analyzed using flow cytometry. (B) Distribution of l-PEVs sizes upon different stimuli. (C) Concentration of α_IIb_β_3_ integrin^+^ l-PEVs was determined using Trucount tubes. Data are mean ± SD; *n* = 12; ∗*P* < .05. (D) Western blotting analysis of extracellular vesicle (EV) markers (CD9) and cell contamination (ER protein calnexin) was performed. Images are representative of *n* = 3. CRP, collagen-related peptide; PLT, platelet.

To generate l-PEVs, platelets were activated with either 20 μM of calcium ionophore A23187 (Sigma Aldrich) or a combination of 1 μg/mL of collagen-related peptide (CRP-A and CRP; Pplus Medical Ltd) and TRAP/PAR-4-activating peptide (CT): 200 μM of SFLLRN-NH2 (Sigma Aldrich) for human or AYPGKF (Cayman Chemical) for murine platelets, respectively. Suspensions were shaken at 1400 rpm for 1 hour at 37 °C. The reaction was stopped by diluting the samples 10 times with Tyrode’s buffer with 2 mM CaCl_2_ (Figure 1A). For experiments with inhibition of metalloproteinases, isolated platelets were pretreated with 100 μM of GM6001 (United States Biological) for 10 minutes at 37 °C.

### 2.4 Analysis of surface GP and glycan profiles using flow cytometry

In Trucount tubes, 5 μL of undiluted sample was combined with 95 μL of Annexin Binding Buffer (BioLegend), stained with CD41-AlexaFluor647 (to detect murine PEVs) or CD61-FITC (to detect human PEVs) and Annexin V-FITC/AlexaFluor647 for 20 minutes at room temperature (RT) (Supplementary Figure 1A, B). PEVs in Trucount tubes were detected based on the expression of CD41 or CD61. Staining with both anti-CD41 and anti-CD61 allowed detection of the platelet population in human and mouse samples (Supplementary Figure 2E, F). After stopping the reaction with 900 μL of Annexin Binding Buffer, 3000 beads were acquired on BD FACSCanto II flow cytometer.

Diluted samples were individually incubated with 5 μg/mL of aforementioned fluorescein-conjugated lectins and antibodies against CD42b, GPVI, and CD62P. Staining was performed for 20 minutes, and reaction was stopped by dilution with Tyrode’s buffer with 2 mM CaCl_2_ filtered through 0.22 μm PES filter; 10,000 events within the vesicles gate were recorded to avoid influence of variability between l-PEVs release from different samples.

Size calibration was performed with Rosetta beads (Exometry). The Mie model was used to calibrate the side scatter (SSC)-A detector and estimate the diameter of round particles [42]. The polystyrene beads (Rosetta beads; Exometry) of known size were recorded at the same settings as the samples of interest. Rosetta calibration software (Exometry) was used to apply Mie model to the SSC detector by recognizing different sizes of Rosetta beads. Through this, the estimations were calculated, and diameter and area of events were applied to the samples of interest. The acquired flow cytometry data were analyzed in FlowJo (FlowJo LLC). The EV gate was defined by setting the upper diameter limit of 1 μm, and the platelet gate was set based on forward scatter vs SSC (Supplementary Figure 1A). Isotype and unstained controls were used for gating of the positive populations of l-PEVs (Supplementary Figure 1B–F). A TritonX-100 control was used to prove that detected events were of biological nature (membrane encapsulated; Supplementary Figure 1G). Lowest detected diameter was 300 nm.

### 2.5 Lectin array in lysates

l-PEVs were generated following CT stimulation and were isolated from supernatant after centrifugation (2500*g* for 15 minutes), which was further filtered through 0.8-μm Nucleopore Track-Etch Membrane filters (Cytiva) to remove remaining cell remnants as described previously [43] (Supplementary Figure 2A). The absence of platelets was confirmed with flow cytometry (Supplementary Figure 2B—filter-isolated PEVs) and western blotting for calnexin (Supplementary Figure 2D). The samples were lysed in RIPA buffer with proteinase and phosphatase inhibitors and stored at −20 °C before assessing an extended panel of carbohydrates using the Lectin Array 95 (RayBiotech Inc; Supplementary Table 1). Analysis of the total lectin content via microarray was performed according to manufacturer’s instructions. Briefly, 1 mg of total protein was labeled with biotin, precipitated on the microarray plate, and labeled with Cy3-streptavidin. The imaging was performed on SureScan Dx Microarray Scanner System (Agilent). The parsing of the spots and assessment of fluorescence of each spot were performed using Python 3.9 (skimage package).

### 2.6 Glycan array analysis

Glycan Array Dashboard (version 3.1) was used to analyze and visualize the glycan array data [44]. The description of the manufacturer regarding lectin binding and the Consortium for Functional Glycomics format was used to draw the structures.

### 2.7 Western blot analysis of EV markers

Lysates of filter-isolated l-PEVs, suspension after stimulation of platelets for 1 hour (platelets and l-PEVs), and isolated resting platelets were prepared in the same manner as for microplate assay, diluted with Laemmli buffer with (for calnexin) or without (for CD9) reducing agent, and boiled for 5 minutes at 95 °C. Furthermore, 10 μg of protein (30 μg of human PEVs) were separated using SDS-PAGE in 10% bis-acrylamide gel and transferred onto 0.22-μm polyvinylidene fluoride membrane. Efficacy of transfer was confirmed with Ponceau S staining (Supplementary Figure 2C, D). Membranes were blocked for 1 hour at RT in Blocking Buffer (15.2 mM Tris-HCl, 4.6 mM Tris [base], 150 mM NaCl, 0.05% Tween20, and 5% bovine serum albumin) and further incubated with primary antibodies against CD9 (Invitrogen; 1:1000) or calnexin (Invitrogen; 1:1000) overnight at 4 °C, followed by 1-hour incubation with horseradish peroxidase-conjugated secondary antibodies (goat anti-rabbit IgG [H + L]; Invitrogen; 1:10,000) at RT. Membranes were developed with SuperSignal West Femto Maximum Sensitivity Substrate (Thermo Scientific) and imaged on a Vilber Lourmat Fusion FX system.

### 2.8 Statistical analyses

Statistical analyses were performed using R software (R Foundation for Statistical Computing). Shapiro–Wilk test was used for analysis of normality of distribution. Outliers were filtered out based on IQR of 25 to 75 criterion. Normally distributed samples were compared using Student *t*-test; nonparametric samples were compared with Mann–Whitney test. A *P* value of <.05 was considered significant.

### 2.9 Data reporting

Data are obtained and reported in line with MISEV2023 guidelines [45], along with MIFlowCyt and MIFlowCyt-EV guidelines [46,47].

## 3 Results

We performed an in-depth analysis of the l-PEV populations generated from human and mouse platelets upon stimulation of collagen receptor (GPVI) and thrombin receptors (PAR1 and PAR4) with CRP and PAR1 (human) or PAR4 (murine)-activating peptide (CT), as well as following incubation with the ionophore A23187 using flow cytometry (Figure 1A). Analysis of the particle size distribution showed that human samples contained more l-PEVs (<1 μm) basally, while murine samples contained very few l-PEVs (Figure 1B). The majority of l-PEVs released from activated platelets were <1 μm in diameter (Figure 1B). Therefore, we gated all particles <1 μm in diameter (Supplementary Figure 1A). Our analysis revealed that murine platelets released less l-PEVs independent of stimulation (Figure 1C). The expression of EV markers in the l-PEV suspension was confirmed using Western blotting (Figure 1D and Supplementary Figure 2C, D). The tetraspanin family protein CD9 was used as an EV marker, while endoplasmic reticulum protein calnexin [48] served as negative control for cell contamination in the EV population [49]. A detergent control was used to confirm that the l-PEVs analyzed were enclosed by a lipid membrane (Supplementary Figure 1G). With this analysis we could show that we were able to isolate l-PEVs and could thus continue our phenotypic comparison between mouse and human l-PEVs.

### 3.1 Phenotyping of GPs on l-PEVs

**Figure 2:**
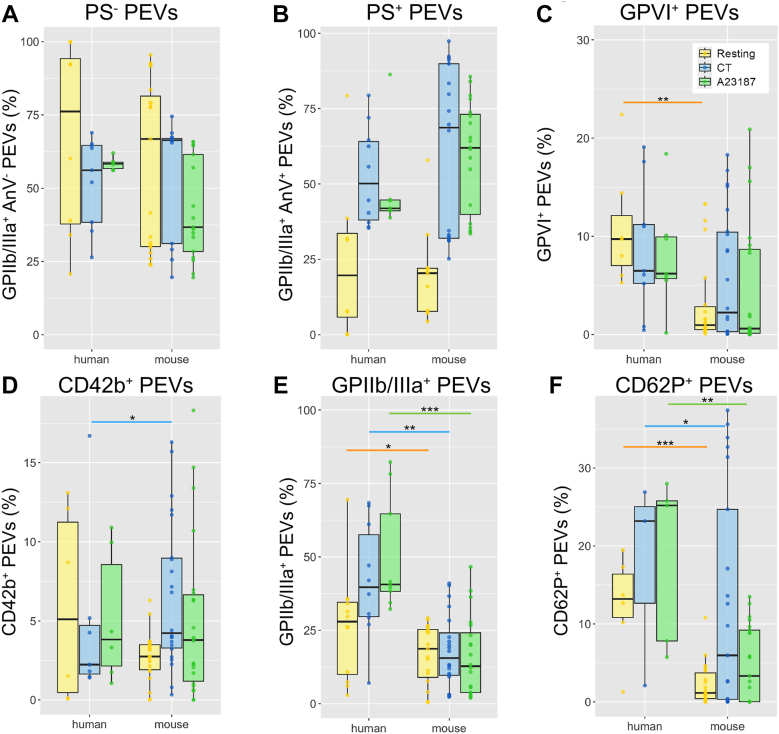
Phenotyping of main platelet glycoproteins (GPs) on large PEVs (l-PEVs). Surface GPs of l-PEVs in unstimulated and activated (CT, A23187) human and murine samples were analyzed using flow cytometry. (A) Percentage of α_IIb_β_3_ integrin^+^ PS^−^ l-PEVs. (B) Percentage of procoagulant (α_IIb_β_3_ integrin^+^ PS^+^) l-PEVs. (C) Percentage of GPVI^+^ l-PEVs. (D) Percentage of CD42b^+^ l-PEVs. (E) Percentage of α_IIb_β_3_ integrin^+^ l-PEVs. (F) Percentage of CD62P^+^ l-PEVs. Data are represented in percent of positive events. Orange, blue, and green lines indicate comparison between human and murine samples in resting, CRP + PAR1/4-AP stimulated (CT), or A23187 stimulated conditions, respectively. Isotype control was used to determine positive populations and set at 1%. Data were calculated after IQR outlier test was performed. Data are mean ± SD; *n* = 6 to 12; ∗*P* < .05, ∗∗*P* < .01, ∗∗∗*P* < .001. PS, phosphatidylserine.

Although human and mouse platelets released procoagulant EVs to a similar extent, mouse platelets tended to generate more PS-positive l-PEVs following stimulation with CT or A23187 (Figure 2A, B). Interestingly, under resting conditions, murine platelet suspensions contained less PS-exposing l-PEVs (Supplementary Figure 3A, B). The proportion of l-PEVs expressing GPVI or CD42b did not exceed 20% (Figure 2C, D), and murine platelets generated more l-PEVs expressing CD42b upon CT stimulation (Figure 2D). Stimulation of human platelets with CT and A23187 (Supplementary Figure 2D-F) resulted in the release of significantly more α_IIb_β_3_-exposing l-PEVs than from murine platelets (Figure 2E). As shown before [24], P-selectin (CD62P, a marker of platelet α-granules) was present on l-PEVs generated by human platelets at higher levels under resting and stimulated conditions compared to murine l-PEVs (Figure 2F). Altogether, these results show distinct types of l-PEVs are being generated from mouse and human platelets, suggesting l-PEVs might interact differently with immune cells between species.

### 3.2 Influence of metalloproteinases on the l-PEV phenotype

**Figure 3:**
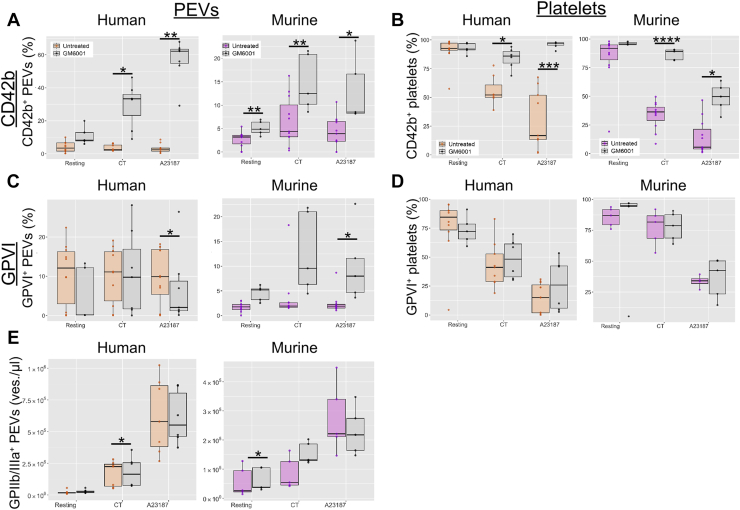
Role of a disintegrin and metalloproteinase 10/17 activity on l-PEV phenotype. The influence of the inhibition of metalloproteinases on platelets and l-PEVs was assessed by pretreatment with pan-MMP inhibitor GM6001 at 100 μM and surface GP profile was analyzed using flow cytometry following activation of mouse and human platelets to induce l-PEV generation. (A) Percentage of CD42b^+^ l-PEVs (B) Percentage of CD42b^+^ platelets. (C) Percentage of GPVI^+^ l-PEVs. (D) Percentage of GPVI^+^ platelets. (E) Concentration of l-PEVs in control and GM6001-treated conditions. Isotype control was set at 1% to determine positive populations. Data are mean ± SD; *n* = 6 (GM6001-treated), *n* = 8 (untreated); ∗*P* < .05, ∗∗*P* < .01, ∗∗∗*P* < .001, ∗∗∗∗*P* < .0001. GP, glycoprotein; PEV, platelet-derived extracellular vesicle.

We found low numbers of l-PEVs expressing GPVI and CD42b (GPIb). Therefore, we pretreated platelets with the metalloproteinase inhibitor GM6001 to decipher if metalloproteinases, such as a disintegrin and metalloproteinase (ADAM) domain 10 (ADAM-10) or ADAM-17, which contribute to GPVI or CD42b shedding, respectively, played a role in GP shedding during l-PEV formation. Metalloproteinase inhibition resulted in a significant increase of CD42b on both human and murine l-PEVs and platelets (Figure 3A, B), whereas GPVI expression was only slightly increased on l-PEVs (Figure 3C, D), suggesting involvement of metalloproteinases in the retention of GPIba and possibly GPVI during l-PEV generation (Figure 3E).

### 3.3 Surface and total glycans on l-PEVs

**Figure 4:**
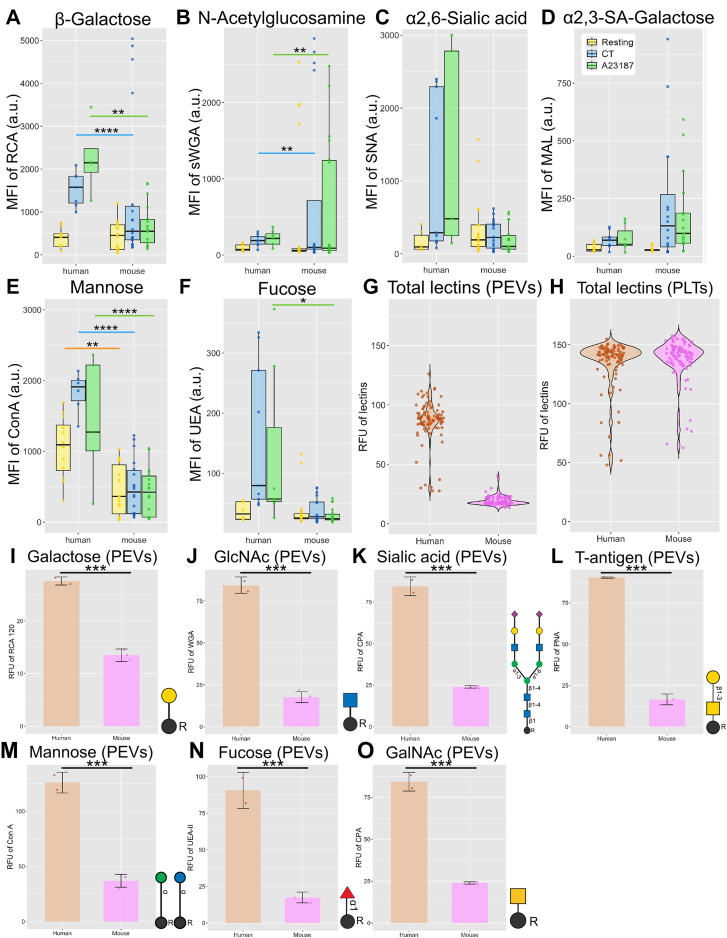
Surface glycans on l-PEVs in human and mice. The binding of fluorescein-conjugated RCA-1 (A), sWGA (B), SNA (C), MAL (D), ConA (E), and UEA lectins (F) to mouse and human l-PEVs was measured using flow cytometry. Data are represented as mean fluorescence intensity (MFI) in arbitrary units (a.u.). Orange, blue, and green lines indicate comparison between human and murine samples in resting, CRP + PAR1/4-AP stimulated (CT), or A23187 stimulated conditions, respectively. Quantification of carbohydrates in lysates of l-PEVs and platelets was performed using a microarray containing 95 lectins. (G) Comparison between total lectin binding to human and murine l-PEVs. (H) Comparison between total lectin binding to human and mouse platelets. Murine l-PEVs contained less carbohydrates, including galactose (I), *N*-acetylglucosamine (J), sialic acid (K), T-antigen core sugars (L), mannose (M), fucose (N), and *N*-acetylgalactosamine (O). Data are represented in relative fluorescence units after normalization of signal on positive and negative controls for protein binding to lectins. Data were obtained after IQR outlier test was performed. Data are mean ± SD; *n* = 8 to 12; ∗*P* < .05, ∗∗*P* < .01, ∗∗∗*P* < .001, ∗∗∗∗*P* < .0001. ConA, concanavalin A; GlcNAc, β-galactose and *N*-acetylglucosamine; MAL, *Maackia amurensis*; PEV, platelet-derived extracellular vesicle; RCA, *Ricinus communis* agglutinin; SNA, *Sambucus nigra*; sWGA, succinylated wheat germ agglutinin; UEA, *Ulex europaeus* agglutinin.

The expression of glycans on the l-PEV surface was analyzed using lectins. We discovered that surface glycans of platelets (Supplementary Figure 5A–F) and l-PEVs (Figure 4A–O and Supplementary Figure 4A–F) underwent significant changes upon stimulation. Surface β-galactose (detected by *R*. *communis* agglutinin 1 lectin) on murine l-PEVs was significantly decreased compared with that on human l-PEVs following stimulation (Figure 4A). GlcNAc exposure (succinylated wheat germ agglutinin) was significantly higher on murine l-PEVs (Figure 4B). Murine l-PEVs showed less surface sialic acid in all conditions tested (Figure 4C and Supplementary Figure 4C), which presumably leads to increased recognition by galactose or β-GlcNAc binding receptors. Mannose (concanavalin A) showed higher expression on the surface of human l-PEVs (Figure 4E). Human l-PEVs (Figure 4F and Supplementary Figure 4F) and platelets (Supplementary Figures 5F and 6F) showed increased fucose residues on their surface.

We then used a lectin microarray to study the complete carbohydrate profiles in l-PEV and platelet lysates. We observed that human and murine platelets showed significantly more lectin binding than their released l-PEVs (Supplementary Figure 8A–F). l-PEVs from mice contained significantly less carbohydrates than l-PEVs derived from humans (Figure 4G), while the carbohydrate content was comparable between murine and human platelets (Figure 4H). The total content of the major monosaccharides fucose, galactose, *N*-acetylgalactosamine, *N*-acetylglucosamine, mannose, sialic acid, and T-antigen was significantly reduced in murine l-PEVs compared with that in human l-PEVs (Figure 4I–O) but not in platelets (Supplementary Figure 7A–G). We discovered that l-PEVs and platelets of both species were enriched with galactose, *N*-acetyllactosamine, *N*-acetylgalactosamine, and mannose (Supplementary Figure 8C–F). High lectin binding to sialic acid was detected in samples from human platelets (Supplementary Figure 8F: PSL1-A and SAMB). Nevertheless, the patterns of top 10 structures in murine and human platelets and l-PEVs remained similar (Supplementary Figure 8C–F). These findings differ from our observations made by flow cytometry but could be explained by the microarray detecting the total content as opposed to the surface glycans.

### 3.4 Influence of handling of platelets on l-PEV phenotype

**Figure 5:**
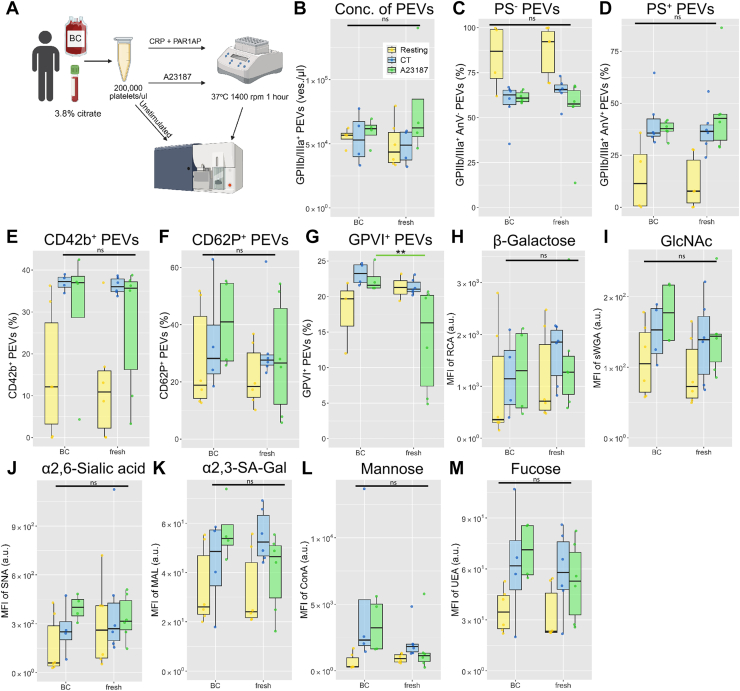
Comparison of l-PEVs generated from BCs- and fresh blood-derived human platelets. (A) Platelets were isolated from either BCs or freshly drawn citrated human whole blood. l-PEVs from unstimulated and activated (CT, A23187) conditions were analyzed using flow cytometry. Concentration of generated l-PEVs (B) and their procoagulant properties (C, D) were assessed in Trucount tubes. (E) Percentage of CD42b^+^ l-PEVs. (F) Percentage of CD62P^+^ l-PEVs. (G) Percentage of l-GPVI^+^ PEVs. Surface binding of RCA-1 (H), sWGA (I), SNA (J), MAL (K), ConA (L), and UEA lectins (M). Green line depicts comparison between A23187-stimulated samples. Data are mean ± SD; *n* = 6; ∗∗*P* < .01. BC, buffy coat; ConA, concanavalin A; CRP, collagen-related peptide; GlcNAc, β-galactose and *N*-acetylglucosamine; MAL, *Maackia amurensis*; MFI, mean fluorescence intensity; ns, not significant; PEV, platelet-derived extracellular vesicle; RCA, *Ricinus communis* agglutinin; SNA, *Sambucus nigra*; sWGA, succinylated wheat germ agglutinin; UEA, *Ulex europaeus* agglutinin.

Since human and murine blood was obtained from different sources, we assessed whether this affected the phenotype of l-PEVs. Human platelets were obtained from BCs prepared on the previous day, while murine platelets were isolated from fresh blood. The BCs were processed within 24 hours, and this time frame is used within blood banks for platelet component manufacturing. Therefore, the platelets are fully viable and functional. Furthermore, we investigated whether storage of platelets in BCs affected the efficacy of vesiculation and phenotype of released l-PEVs. Comparison of platelets isolated from BCs and fresh blood did not reveal any major differences between phenotype of l-PEVs (Figure 5A–M) and platelets (Supplementary Figure 9A–I).

**Figure 6:**
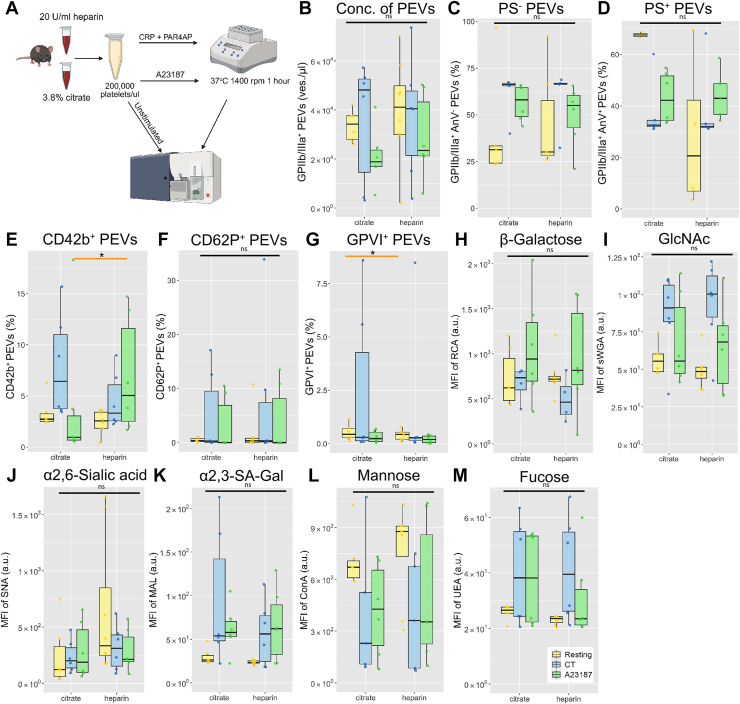
Comparison of l-PEVs generated from platelets isolated from citrated or heparinized murine blood. (A) Platelets were isolated from citrated or heparinized murine whole blood. l-PEVs from unstimulated and activated (CT, A23187) conditions were analyzed using flow cytometry. Concentration of generated l-PEVs (B) and their procoagulant properties (C, D) were assessed in Trucount tubes. (E) Percentage of CD42b^+^ l-PEVs. (F) Percentage of CD62P^+^ l-PEVs. (G) Percentage of GPVI^+^ l-PEVs. Surface binding of RCA-1 (H), sWGA (I), SNA (J), MAL (K), ConA (L), and UEA lectins (M). Orange line depicts the comparison between resting samples. Data are mean ± SD; *n* = 6; ∗*P* < .05. ConA, concanavalin A; CRP, collagen-related peptide; GlcNAc, β-galactose and *N*-acetylglucosamine; MAL, *Maackia amurensis*; MFI, mean fluorescence intensity; ns, not significant; PEV, platelet-derived extracellular vesicle; RCA, *Ricinus communis* agglutinin; SNA, *Sambucus nigra*; sWGA, succinylated wheat germ agglutinin; UEA, *Ulex europaeus* agglutinin.

Murine platelets were isolated from whole blood anticoagulated with sodium heparin, while sodium citrate was used as an anticoagulant in human samples. Therefore, we analyzed l-PEVs generated from murine platelets that were isolated from citrated or heparinized whole blood (Figure 6A–M and Supplementary Figure 10A–L). Similarly, no major differences between the different anticoagulants used were revealed.

## 4 Discussion

We performed analyses of l-PEVs generated from mice and human platelets and studied their phenotypes to clarify to which extent human and mouse l-PEVs are comparable. We confirmed previously shown distribution of main human platelet GPs (CD42b, GPVI, CD62P, and integrin α_IIb_β_3_) on l-PEVs, generated following 2 types of stimulations (ionophore treatment and physiological stimulation via collagen and thrombin receptors). The comparison of the carbohydrate structures on platelets and l-PEVs revealed principal interspecies differences. For the first time, mouse l-PEV are compared directly with their human counterparts.

Glycan structures can be simple *O*-linked or more complex *N*-linked, most are capped by sialic acid. Platelets are highly glycosylated, especially CD42b, which is predominantly *O*-glycosylated [50]. We confirmed previous findings that platelet activation leads to removal of sialic acid [15,51]. Rosenbalm et al. [52] revealed diverse *N*-glycans, including high mannose structures, complex glycans, and blood group-related glycans. *O*-glycan largely comprised sialylated and fucosylated core-1 and core-2 structures. Platelets are activated during their storage, and both *N*- and *O*-linked glycans were desialylated [52]. More in-depth mass spectrometry of the platelet glycome showed previously undescribed complex structures, which were highly variable between healthy individuals and unique. Platelets were sialylated and desialylated during storage and glycan structures were high in mannose, contained animal diet-derived sialic acid type N-glycolylneuraminic acid, and consisted of unique complex Lewis X structures [53].

Recent findings showed that when human platelets were activated with thrombin, the releasate containing a plethora of PEVs contained *O*-fucosylation on multimerin-1, an α-granule protein that supports platelet adhesion to collagen and is a carrier for factor V [54]. In this study, we used native samples with a mixture of platelets and l-PEVs to avoid changing the phenotype of the particles by use of common EV isolation techniques, that is, centrifugation and ultracentrifugation [20], size-exclusion chromatography, or size filtration.

We compared the phenotypes of l-PEVs derived from murine and human platelets and found distinct characteristics for each of the 2 species. We confirmed the already described phenotypes of the l-PEVs (presence of main platelet markers on PEVs) [23,24,55]. As expected, platelets generated many procoagulant (PS^+^) l-PEVs that contribute to enhancement of thrombosis [56]. Furthermore, the expression of main platelet GPs was different between mice and humans: P-selectin and α_IIb_β_3_ integrin were significantly diminished on murine l-PEVs compared with those on human l-PEVs, unlike in other studies [57].

Most studies investigate procoagulant (α_IIb_β_3_ integrin^+^ PS^+^) PEVs [56]. It has been shown that platelet stimulation with different agonists leads to the generation of distinct l-PEV phenotypes. For instance, binding of integrin α_IIb_β_3_ integrin to fibrinogen leads to the generation of larger PEVs (>100 nm), while stimulation of GPVI with CRP results in the release of small PEVs [8]. Protein cargo and RNA loading of PEVs also depend on the type of stimulation: GPVI-mediated stimulation of platelets generate PEVs enriched with interleukin 6 and TWEAK; GPVI/PAR stimulation results in enrichment of PEVs with CXCL28 and CXCL29 [58].

In both species, surface GPVI and CD42b (GPIb) on l-PEVs were low (eg, the proportion of CD42b^+^ l-PEVs from both species never exceeded 20%). The low expression of CD42b on l-PEVs was restored upon pretreatment of platelets with the matrix metalloproteinase inhibitor GM6001. Interestingly, the GPVI expression on l-PEVs was only affected to some extent. In platelets, ADAMs cleave the extracellular part of GPs during activation [59]. Catalytically active ADAMs can be present in PEVs as well [60]. It was previously shown that ADAM-10 activity in platelets is increased upon shear-stressed conditions but not upon stimulation of platelets with conventional GPVI agonists (convulxin, CRP, and collagen) [61] under shaking conditions [62]. This might explain the absence of GPVI on l-PEVs even upon treatment with GM6001. Increased CD42b expression could play a role in the recruitment of PEVs by monocytes and their adhesion to endothelial cells [32].

We observed distinct expression patterns not only of surface GPs but also of carbohydrates on l-PEVs from human and murine samples. This is relevant since EV glycans plays an important role for the recognition of EVs by various cell types (hepatocytes, fibroblasts, and neurons) [55,63,64]. A previous study on human PEVs generated during platelet storage showed that most glycans were altered on the procoagulant PS^+^ PEV subpopulation [20]. In our study, surface β-galactose on murine l-PEVs was significantly decreased compared with human l-PEVs, which could lead to less interaction, for example, with macrophage galactose lectin on phagocytes [65]. Surface mannose was higher in human l-PEVs, which could improve binding to the mannose receptor (CD206) on macrophages [66]. Murine platelets and l-PEVs also showed significantly lower fucose exposure than human samples.

Computational analysis was earlier performed to assess the role of glycans and specific nodes on the EVs derived from metastatic cells. Analysis revealed high enrichment with glycan nodes responsible for adhesion of EVs to extracellular matrix proteins in bone marrow and lungs (2,4-mannose that represents β1,4-branching of N-glycans) [63]. Therefore, understanding the glycan structure could provide insights into the targeted delivery of nanoparticle type therapeutics.

We also observed that the total carbohydrate content differed from the surface exposure. Although we used a common approach for the analysis of carbohydrates with microarray [67] and flow cytometry using lectins [15,20,51,[68], [69], [70]], mass spectrometry remains the gold standard for more detailed studies of the glycan structures [53,71].

Moreover, the lectins used might have several specificities, for instance, *S*. *nigra* lectin binds to a variety of carbohydrates, including lactose, galactosamine, galactose, and fucose [[72], [73], [74]]. Mass spectrometry of PEVs would be of able to decipher the carbohydrate structures in detail.

Platelet biodistribution is highly dependent on their surface receptor phenotype. For instance, platelets treated with exogenous enzymes that cleave sialic acid, neuraminidase, accumulate in the liver to be removed from the bloodstream [16,75]. A high surface expression of P-selectin (CD62P) would enhance interactions between platelets and immune cells that express PSGL-1 [76], including myeloid and lymphoid cells. Studying the glycan profile of the PEV membrane is of high interest due to the dependency of biodistribution on the carbohydrate exposure [21,55,63]. Studies of EV biodistribution conclude that main organs of accumulation of microparticles are the liver, spleen, and kidneys [36]. So far, only PS-mediated clearance has been analyzed; however, the majority of EVs are PS-negative [20], thus suggesting a closer look at the function and clearance of nonprocoagulant PEVs.

Despite the different preprocessing of the platelet-containing samples during initial analysis, we confirmed that the storage of platelets in BCs for 1 day does not affect their ability to generate l-PEVs compared with platelets isolated from freshly drawn blood. The surface phenotype of generated l-PEVs was also not affected by the storage. We also compared standard anticoagulation agents for blood withdrawal from mice. Trivigno et al. [77] provided evidence that the anticoagulant does not affect platelet function, and our data add to the knowledge that l-PEVs generation is also not affected by either.

Despite the discovered differences between murine and human l-PEVs, our research is limited by the resolution of the flow cytometer used (BD FACSCanto II; minimal size detected = 300 nm). An imaging flow cytometry and use of high-resolution approaches for EV detection and characterization (nanoparticle tracking analysis, microscopy, and imaging flow cytometry [48]) would allow to better analyze the size distribution of the generated particles and potentially reveal subsets we did not observe (CD62P^+^ PEVs and small EVs [8]). On the contrary, the phenotyping was conducted without isolating the PEVs to ensure intact surface glycans as they are highly sensitive to shear forces [20]. Therefore, the observed surface distribution of the glycans resembles the properties of l-PEVs generated in (patho)physiology.

We discovered significant differences between l-PEVs generated from human and murine platelets providing important knowledge for future PEV biodistribution studies in animal models of disease. Translating results from PEV *in vivo* studies in mice to humans should therefore be considered cautiously. Since platelet components contain a plethora of PEVs [78], platelet transfusion leads to a massive exposure of patients to PEVs. Therefore, studies of biodistribution of specifically PEVs is of high interest for the improvement of storage of platelet products for transfusion. It was shown that transfused PEVs interact with endothelial cells in the vasculature [79] of lungs [80], accumulate in the liver and spleen [2], and get consumed by phagocytes in the circulation [81]. So far, little is known about the consequences of this massive PEV uptake. Thus, future clinical trials and animal studies will provide more clues on PEV interactions with immune cells and homing and/or clearance in recipients. Studying the biodistribution of large and small EV is important in the development of diagnostic tools and novel therapeutic options. PEVs contribute to various diseases (cardiovascular, trauma-induced coagulopathy, disseminated intravascular coagulation, and cancer). Therefore, investigating the specific interactions of PEVs with immune cells will allow us to study the changes induced on immune cells by PEVs.
