# Supplementary material for: Comparative phenotyping of surface markers and glycans in murine and human platelet-derived extracellular vesicles
Source: Res Pract Thromb Haemost. 2026 Mar 20;10(3):103414. doi: 10.1016/j.rpth.2026.103414 (PMC13092044; doi:10.1016/j.rpth.2026.103414)
Supplement: Supplementary Material [file mmc1.pdf]

Supplementary Information

Phenotyping murine and human platelet-derived extracellular vesicles gives implications for future in vivo biodistribution and clearance studies

Olga An[1], Friedrich Reusswig[1], Viola Krenzlin[1], Carsten Deppermann*[1,2] and Dianne E. van der Wal*[3,4]

Affiliations:

1. Center for Thrombosis and Hemostasis, University Medical Center of the Johannes Gutenberg University Mainz, Mainz, Germany
2. Research Center for Immunotherapy, University Medical Center of the Johannes Gutenberg University Mainz, Mainz, Germany
3. ANZAC Research Institute, Concord Repatriation Hospital, Australia
4. School of Medical Sciences, Faculty of Medicine and Health, University of Sydney, Sydney, Australia

*contributed equally to the paper

MISEV2018 Checklist

Sections are referred to the sections listed in: J.A. Welsh et al, " Minimal information for studies of extracellular vesicles (MISEV2023): From basic to advanced approaches", J Extracell Vesicles 2024;13; e12404.

1-Nomenclature

The extracellular vesicles derived from platelets are abbreviated as l-PEVs (large PEVs). The l-PEVs are defined as round events smaller than 1 µm in diameter according to the calibration of the samples with use of Rosetta beads (Fig. 1A). Platelets are out-gated because of inapplicability of Mie theory to particles of irregular shape.

2-Collection and pre-processing

Human samples

Human platelets were isolated from buffy coats received from the Transfusion Center of the University Medical Center Mainz. The buffy coats were received the next day after the blood donation (storage at RT, within 24 hours).

The buffy coats were collected from healthy adult donors (≥18 years) eligible for donation of blood for transfusions. The donors had no infectious, cardiac, lung, liver, kidney, spleen, neurological, blood, immune disorders; did not intake medications (retinoids, antibiotics, NSAIDs, injections including dermatologic, e.g., Botox).

9 ml out of 150 mL available was used for platelet isolation. Platelets were isolated with three sequential centrifugations, all in presence of 3.8% sodium citrate: 100g 8 minutes, twice 400g for 5 minutes.

For experiments with fresh blood-derived platelets, 10 ml of peripheral blood was collected into 3.8% sodium citrate-containing vacuum tubes (Sarstedt, Germany) from healthy adult donors (≥18 years) eligible for blood donation (ethics committee of the state medical association of Rhineland-Palatinate vote № 2024-17880_1). The donors had no acute infectious, cardiac, lung, liver, kidney, spleen, neurological, blood, immune disorders; did not take medications (retinoids, antibiotics, non-steroid anti-inflammatory drugs (NSAIDs), injections including dermatologic, e.g., Botox). Prior to filling the sample tube, a waste tube was used to discard the first portion of blood to avoid the influence of tissue factor contamination on platelet function. Tubes with blood were left at 37°C for 20 minutes after blood withdrawal for desensitization of platelets from shear stress. Further processing was performed as for buffy coats.

Murine samples

C57BL/6 mice of 10-12 weeks were bled via retroorbital sinus with hematocrit capillaries pre-coated with heparin (Leo Pharma, Neu-Isenburg, Germany) into heparin-containing tubes. 700 µl of blood were collected. Whole blood was centrifuged in presence of 10 U/mL of heparin at 60g for 6 minutes. PRP was centrifuged in presence of 10 U/mL of heparin at 60g for 6 minutes to remove contaminating red blood cells. Platelets were obtained after centrifugation of PRP at 650g for 5 minutes in presence of 1 U/mL of apyrase (Sigma Aldrich, St. Louis, USA) and 0.5 µM of prostacyclin (Cayman Chemical, Ann Arbor, USA). Pellet was resuspended in Tyrode's buffer (140 mM NaCl, 0.5 mM $NaHCO_3$, 3 mM KCl, 0.5 mM $MgCl_2$, 10 mM D-glucose, 10 mM HEPES, pH 7.35) with 2 mM of $CaCl_2$ filtered through 0.22 µm PES filter (Carl Roth GmbH, Karlsruhe, Germany).

All centrifugation steps were performed at room temperature using an Eppendorf 5425 centrifuge (rotor FA-24x2, acceleration 9, deceleration 6) or Eppendorf 5910 Ri centrifuge (rotor S-4xUniversal, acceleration 6, no break). All the additives to the blood and blood components were pre-warmed to 37°C.

The influence of anticoagulant was also assessed and murine blood was obtained in the same manner as human whole blood, however in presence of sodium citrate instead of sodium heparin.

Generation of l-PEVs

Isolated platelets from both human and mice were concentrated to 200 x 10^6/mL. l-PEVs were generated using two approaches: stimulation of thrombin and collagen receptors with respective agonists (200 µM of SFLLRN (Sigma Aldrich, St. Louis, USA) for human or AYPGKF (Cayman Chemical, Ann Arbor, USA) for murine platelets + 1 µg/mL of collagen-related peptide – CRP (CRP-A, pPlusMedical Ltd., Dublin, Ireland)) and treatment of platelets with 20 µM of calcium ionophore A23187 (Sigma Aldrich, St. Louis, USA). Activated platelets were shaken for 1 hour at 1350 rpm at 37 ^{o}C. After generation of l-PEVs the suspensions were diluted 10 times with Tyrode's buffer with 2 mM $CaCl_2$, filtered through 0,22µm PES sterile filter (Carl Roth GmbH, Karlsruhe, Germany).

3-EV separation and concentration

Analysis of the phenotype of the l-PEVs was performed without isolating the l-PEVs from remaining platelets to prevent possible phenotypical and glycan changes upon isolation due to centrifugal forces [1].

The Lectin array 95 (RayBiotech Inc., GA, USA) was performed on the isolated l-PEVs and isolated platelets from the suspensions after generation of the l-PEVs. The suspensions were centrifuged in

presence of 3.8% of sodium citrate at 2500g for 15 minutes, the top 80% of the supernatant were collected and centrifuged in presence of 3.8% of sodium citrate at 2500g for 15 minutes. Top 80% of the supernatant were collected and filtered through 0.8 µm Nucleopore™ Track-Etch Membrane filters (Cytiva, NA, USA) [2]. The lack of platelet contamination was confirmed by flow cytometry (Fig. S2B). Platelet pellet was also collected for analysis of glycans. The l-PEVs and platelet pellets were incubated in RIPA buffer with inhibitors of proteinases (cOmplete Protease Inhibitor Cocktail, Roche, MA, USA) and phosphatases (phosStop, Roche, MA, USA) for 30 minutes and frozen at -20 ^{o}C for later analysis.

The recovery of l-PEVs was confirmed with flow cytometry using Trucount™ tubes (see section 4).

4-EV characterization

Quantification

Number of l-PEVs in the samples was calculated using Trucount™ tubes (BD, New Jersey, USA). 5 µl of undiluted suspensions of platelets and l-PEVs after 1 hour of generation of l-PEVs was added to 95 µL of Annexin Binding buffer (BioLegend, San Diego, USA), stained with CD41-AlexaFluor 647 (anti-mouse) or CD61-FITC (anti-human) and AnnexinV-AlexaFluor 647 for 20 minutes at RT. The reaction was stopped with 900 µL of Annexin Binding buffer. 3,000 events in Trucount™ beads were recorded, and the concentration of PEVs was calculated.

Protein concentration for the Lectin array 95 was determined with DC assay (Bio-Rad, CA, USA). The proteins were concentrated to 1 mg/mL according to manufacturer's instructions for the Lectin array using Amicon Ultra Centrifugal filters (30 kDA MWCO, Merck Millipore, MA, USA).

Global characterization

Western blotting

The filter-isolated l-PEVs, the isolated platelets and suspension after 1 hour of platelet stimulation were incubated in RIPA buffer with inhibitors of proteinases and phosphatases for 30 minutes, diluted with Laemlli Sample buffer with reducing agent (for calnexin) or without reducing agent (for CD9) [3] and boiled at 95°C for 5 minutes followed by instant freezing at -20 ^{o}C. 10 µg of proteins from each suspension, except for human l-PEVs (30 µg was taken) were loaded into 10% bis-acrylamide gel and SDS-PAGE was performed to separate proteins. Proteins were transferred onto 0.22 µm PVDF membranes using semi-dry blotting. Membranes were blocked for 1 hour at room temperature in Blocking Buffer (15.2 mM Tris-HCl, 4.6 mM Tris (base), 150 mM NaCl, 0.05% Tween20, 5% BSA). Membrane with reduced samples were incubated with 1:1000 of anti-calnexin polyclonal

antibody (#PA5-34754, Invitrogen, MA, USA), non-reduced membranes were incubated with 1:1000 of anti-CD9 antibody (#MA5-32333, Invitrogen, MA, USA). Incubation with primary antibodies was performed at 4 ^{o}C overnight. Further, membranes were incubated with 1:10,000 of anti-goat HRP-conjugated secondary antibodies (IgG (H + L), #31466, Invitrogen, MA, USA) for 1 hour at room temperature. Membranes were developed with SuperSignal^{TM} West Femto Maximum Sensitivity Substrate (Thermo Scientific, MA, USA) and imaged on a Vilber Lourmat Fusion FX system.

Flow cytometry

The surface phenotype of the l-PEVs was assessed by flow cytometry. Resting, unstimulated shaken, CRP + PAR1/4 stimulated, A23187 stimulated samples were incubated with antibodies against main platelet glycoproteins (5% of CD42b, 5% of GPVI, 5% of CD62P, 2% of CD41 or 2% of CD61) and 5 µg/mL of lectins (*Ricinus communis* agglutinin, succinylated *Wheat Germ* agglutinin, Sambucus nigra lectin, *Maackia amurensis* lectin, Concanavalin A, *Ulex europaeus* agglutinin) for 20 minutes at RT. The reaction was stopped with 10 times dilution with filtered (0,22 µm PES sterile filters) Tyrode's buffer with 2 mM $CaCl_2$.

Fluorescence was detected in BD FACSCanto II (BD, New Jersey, USA). Measurements were performed at low (10 µl/min) or medium (60 µl/min) flow rate with target speed range of 200-900 events/s. 10,000 events in l-PEV gate were recorded to avoid influence of variability between l-PEVs release from different samples. Buffer control, unstained control and isotype controls were used for determination of target populations. Calibration on size was performed with Rosetta beads (Exometry, Amsterdam, the Netherlands). Mie theory was applied to SSC-A detector to estimate the real diameters of the l-PEVs. Manufacturer's software was used to calibrate the samples [4].

Lectin-detection microarray

Assessment of total carbohydrate content of l-PEVs and platelets was performed with the Lectin array 95. 1 mg of protein was loaded onto the microarray plate and processed according to manufacturer's instructions. The imaging was performed on SureScan Dx Microarray Scanner System (Agilent, CA, USA). The spots were parsed and the fluorescence of each spot was derived using Python 3.9 (skimage package). Results were visualized in the GLAD tool [5].

5 - Technique-specific reporting for EV characterization

Flow cytometry was performed and reported according to MIFlow-Cyt and MIFlow-CytEV. Checklists are provided below.

6 – EV release and uptake

N/A

7-Functional studies

N/A

8 – EV analysis in vivo

N/A

9-Reporting

Data is available under reasonable request from the corresponding author.

References

[1] Van Der Wal DE, Rey Gomez LM, Hueneburg T, Linnane C, Marks DC. Changes in glycans on platelet microparticles released during storage of apheresis platelets are associated with phosphatidylserine externalization and phagocytosis. Transfusion 2022;62:1289–301. https://doi.org/10.1111/trf.16891.

[2] Bettin B, Gasecka A, Li B, Dhondt B, Hendrix A, Nieuwland R, et al. Removal of platelets from blood plasma to improve the quality of extracellular vesicle research. Journal of Thrombosis and Haemostasis 2022;20:2679–85. https://doi.org/10.1111/jth.15867.

[3] Welsh JA, Goberdhan DCI, O'Driscoll L, Buzas EI, Blenkiron C, Bussolati B, et al. Minimal information for studies of extracellular vesicles (MISEV2023): From basic to advanced approaches. J of Extracellular Vesicle 2024;13:e12404. https://doi.org/10.1002/jev2.12404.

[4] De Rond L, Coumans FAW, Nieuwland R, Van Leeuwen TG, Van Der Pol E. Deriving Extracellular Vesicle Size From Scatter Intensities Measured by Flow Cytometry. CP Cytometry 2018;86:e43. https://doi.org/10.1002/cpcy.43.

[5] Mehta AY, Cummings RD. GLAD: GLycan Array Dashboard, a visual analytics tool for glycan microarrays. Bioinformatics 2019;35:3536–7. https://doi.org/10.1093/bioinformatics/btz075.

Figure Legends

Figure S1. Gating strategy of human and murine platelets and PEVs.

A. Gating strategy including size gating of l-PEV and platelets on FSC *vs* SSC, followed by sub-gating of l-PEV on different sizes. Events smaller than 1 µm in diameter were gated as l-PEVs. Positive populations of l-PEV were derived from isotype controls for respective antibodies or unstained sample. B. Gating of $CD41^+$ or $CD61^+$ l-PEVs, followed by definition of PS exposing and non-exposing l-PEVs. C. Gating of $lectin^+$ l-PEVs, D. Gating of $GPVI^+$ l-PEVs. E. Gating of $CD42b^+$ l-PEVs. F. Gating of $CD62P^+$ l-PEVs. **G.** Triton control was used to confirm biological nature (membrane enclosed) of analyzed EV.

Figure S2. Methodological controls.

A. Schematic description of l-PEV isolation. **B.** Representative flow cytometry plots of samples used for Western blots. **C.** Western blots for CD9. **D.** Western blots for calnexin. **E.** Comparison of CD41 and CD61 for detecting murine l-PEVs and platelets (PLTs). **F.** Comparison of CD41 and CD61 staining to identify murine and human platelets (PLTs). DP – positive for both CD61 and CD41. Mean ± SD; n = 3.

Figure S3. Characteristics of human and murine platelets and l-PEVs

Flow cytometric analysis of resting and stimulated platelets and l-PEVs was performed. **A.** Percentage of $\alpha_{IIb}\beta_3$ integrin $^+$ PS^- l-PEVs. **B.** Percentage of $\alpha_{IIb}\beta_3$ integrin $^+$ PS^+ l-PEVs. **C.** Concentration of $\alpha_{IIb}\beta_3$ integrin $^+$ PS^- platelets. **D.** Concentration of $GPIIb/IIIa^+$ PS^+ platelets. **E.** Percentage of $GPVI^+$ platelets. **F.** Percentage of $CD42b^+$ platelets. **G.** Percentage of $\alpha_{IIb}\beta_3$ integrin $^+$ platelets. **H.** Percentage of $CD62P^+$ platelets. Data is represented in concentration (A, B) and percent of positive events (C-F). Orange, blue or green lines indicate the comparison between human and murine samples in resting, CRP+PAR1/4-AP stimulated or A23187 stimulated conditions. Isotype control was used to determine positive populations. * - p-value <0.05, ** - p-value <0.01, *** - p-value <0.001, **** - p-value <0.0001.

Figure S4. Surface expression of carbohydrates on l-PEVs.

The binding of RCA-1 (**A**), sWGA (**B**), SNA (**C**), MAL (**D**), ConA (**E**) and UEA (**F**) to l-PEVs under resting and activated conditions was analyzed by flow cytometry to determine the exposure of various glycans. Percent of l-PEVs stained with lectins are plotted. Orange, blue or green lines indicate the

comparison between human and murine samples in resting, CRP+PAR1/4-AP stimulated or A23187 stimulated conditions. * - p-value <0.05, ** - p-value <0.01, *** - p-value <0.001, **** - p-value <0.0001.

Figure S5. Surface expression of carbohydrates on platelets.

The binding of RCA-1 (**A**), sWGA (**B**), SNA (**C**), MAL (**D**), ConA (**E**) and UEA (**F**) to platelets under resting and activated conditions was measured by flow cytometry to determine the exposure of various glycans. Mean fluorescence intensities of lectin binding to platelets are plotted. Orange, blue or green lines indicate the comparison between human and murine samples in resting, CRP+PAR1/4-AP stimulated or A23187 stimulated conditions. * - p-value <0.05, ** - p-value <0.01, *** - p-value <0.001, **** - p-value <0.0001.

Figure S6. Surface glycans on human and murine platelets.

The binding of RCA-1 (**A**), sWGA (**B**), SNA (**C**), MAL (**D**), ConA (**E**) and UEA (**F**) to platelets under resting and activated conditions was measured by flow cytometry to determine the exposure of various glycans. The positive population was determined based on the unstained control. The flow cytometry data is represented in percent of positive platelets for a respective lectin. Orange, blue or green lines indicate the comparison between human and murine samples in resting, CRP+PAR1/4-AP stimulated or A23187 stimulated conditions. * - p-value <0.05, ** - p-value <0.01, *** - p-value <0.001, **** - p-value <0.0001.

Figure S7. Expression of major groups of glycans in platelets.

Major groups of carbohydrates, including fucose (**A**), galactose (**B**), N-Acetylgalactosamine (**C**), N-Acetylglucosamine (**D**), mannose (**E**), T-antigen (**F**), sialic acid (**G**) were detected with lectin microarray. Data is represented in relative fluorescence unit derived from normalization of the values to positive and negative controls for protein binding to lectins. Heatmaps represent differences between lectin binding to human and murine l-PEVs (**H**) and platelets (**I**)**.** Ratios between average human RFUs to murine RFUs are indicated by color-coding. p-values are given for each (bold represents p<0.05).

Figure S8. Total carbohydrate content in murine and human platelets and l-PEVs.

The expression of carbohydrates was detected using lectin microarray. Both murine (**A**) and human (**B**) platelets showed increased enrichment in glycans compared to their l-PEVs. The top 10 binding lectins are shown for murine (**C**) and human (**D**) PEVs along with murine (**E**) and human (**F**) platelets. Data is represented in relative fluorescence unit derived from normalization of the values to positive and negative controls for protein binding to lectins.

Figure S9. Characterization of platelets isolated from buffy coats and freshly drawn blood.

Flow cytometric analysis of resting and stimulated platelets and l-PEVs derived from buffy coats or freshly drawn peripheral blood was performed. **A**. Concentration of platelets. **B**. Concentration of $\alpha_{IIb}\beta_3$ integrin $^+$ PS^- platelets. **C.** Concentration of $\alpha_{IIb}\beta_3$ integrin $^+$ PS^+ platelets. **D**. Percentage of CD42b+ platelets. **E**. Percentage of CD62P+ platelets. **F**. Percentage of $GPVI^+$ platelets. The binding of RCA-1 (**G**), sWGA (**H**), SNA (**I**), MAL (**J**), ConA (**K**) and UEA (**L**) to platelets in mean fluorescence intensity in arbitrary units (a.u.). Mean ± SD; n = 6.

Figure S10. Characterization of platelets isolated from citrated and heparinized blood.

Flow cytometric analysis of resting and stimulated platelets and l-PEVs derived from citrated or heparinized whole blood was performed. **A**. Concentration of platelets. **B**. Concentration of $\alpha_{IIb}\beta_3$ integrin $^+$ PS^- platelets. **C.** Concentration of $\alpha_{IIb}\beta_3$ integrin $^+$ PS^+ platelets. **D**. Percentage of CD42b+ platelets. **E**. Percentage of CD62P+ platelets. **F**. Percentage of $GPVI^+$ platelets. The binding of RCA-1 (**G**), sWGA (**H**), SNA (**I**), MAL (**J**), ConA (**K**) and UEA (**L**) to platelets in mean fluorescence intensity in arbitrary units (a.u.). Mean ± SD; n = 6.

Figure S1

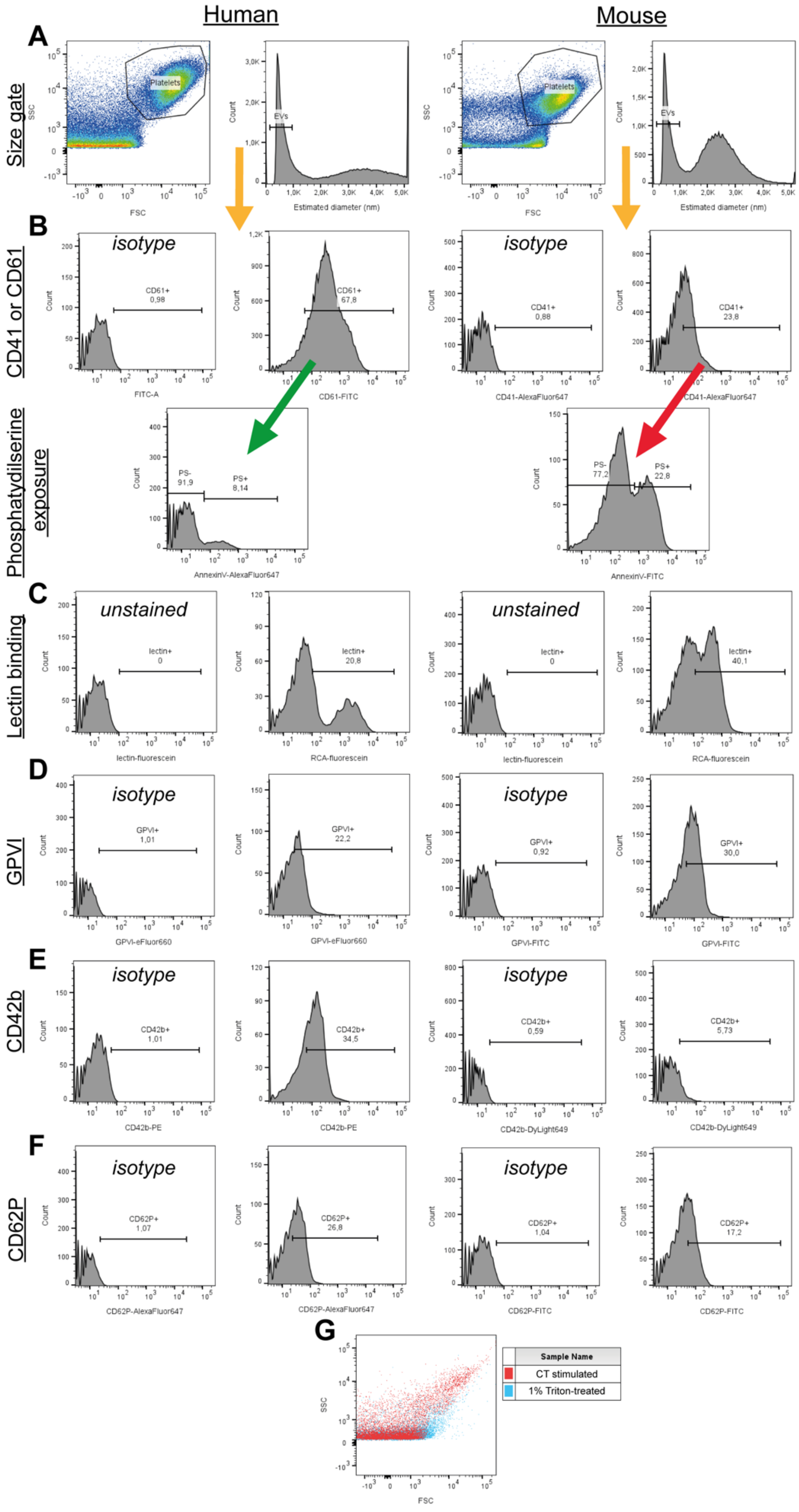
Human
Mouse
A
Size gate
Platelets
EVs
SSC
FSC
Count
Estimated diameter (nm)
B
CD41 or CD61
isotype
CD61+
0,98
67,8
FITC-A
CD61-FITC
CD41+
0,88
23,8
CD41-AlexaFluor647
Phosphatydilserine exposure
PS-
91,9
PS+
8,14
AnnexinV-AlexaFluor647
PS-
77,2
PS+
22,8
AnnexinV-FITC
C
Lectin binding
unstained
lectin+
0
20,8
40,1
lectin-fluorescein
RCA-fluorescein
D
GPVI
GPVI+
1,01
22,2
0,92
30,0
GPVI-eFluor660
GPVI-FITC
E
CD42b
CD42b+
1,01
34,5
0,59
5,73
CD42b-PE
CD42b-DyLight649
F
CD62P
CD62P+
1,07
26,8
1,04
17,2
CD62P-AlexaFluor647
CD62P-FITC
G
Sample Name
CT stimulated
1% Triton-treated

Figure S2

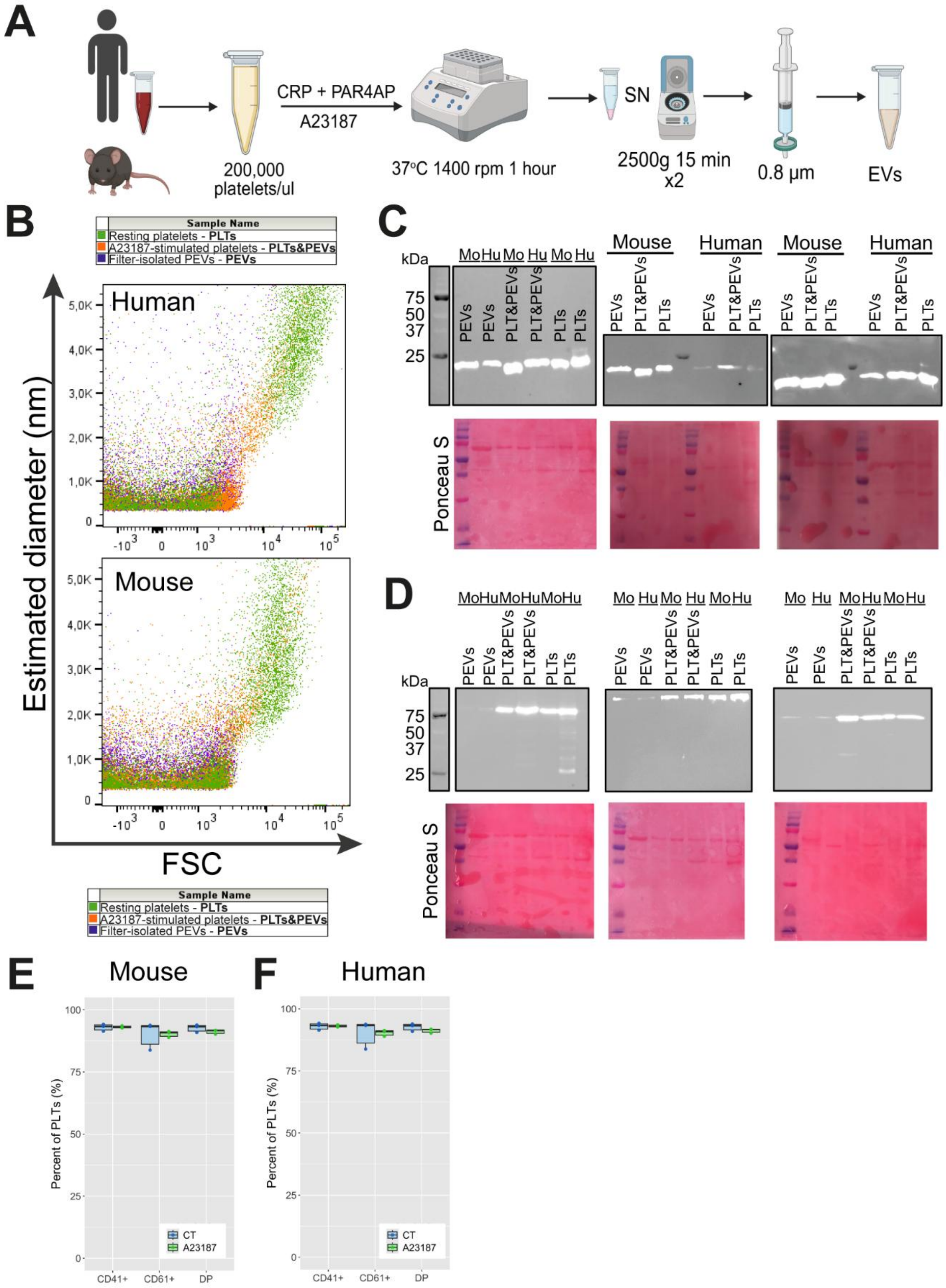
A
CRP + PAR4AP
A23187
200,000 platelets/ul
37°C 1400 rpm 1 hour
SN
2500g 15 min x2
0.8 µm
EVs
B
Sample Name
Resting platelets - PLTs
A23187-stimulated platelets - PLTs&PEVs
Filter-isolated PEVs - PEVs
Human
Mouse
Estimated diameter (nm)
FSC
C
kDa
75
50
37
25
Mo Hu Mo Hu Mo Hu
PEVs
PLT&PEVs
PLTs
Mouse
Human
Ponceau S
D
Mo Hu Mo Hu Mo Hu
E
Mouse
F
Human
Percent of PLTs (%)
CT
A23187
CD41+
CD61+
DP

Figure S3

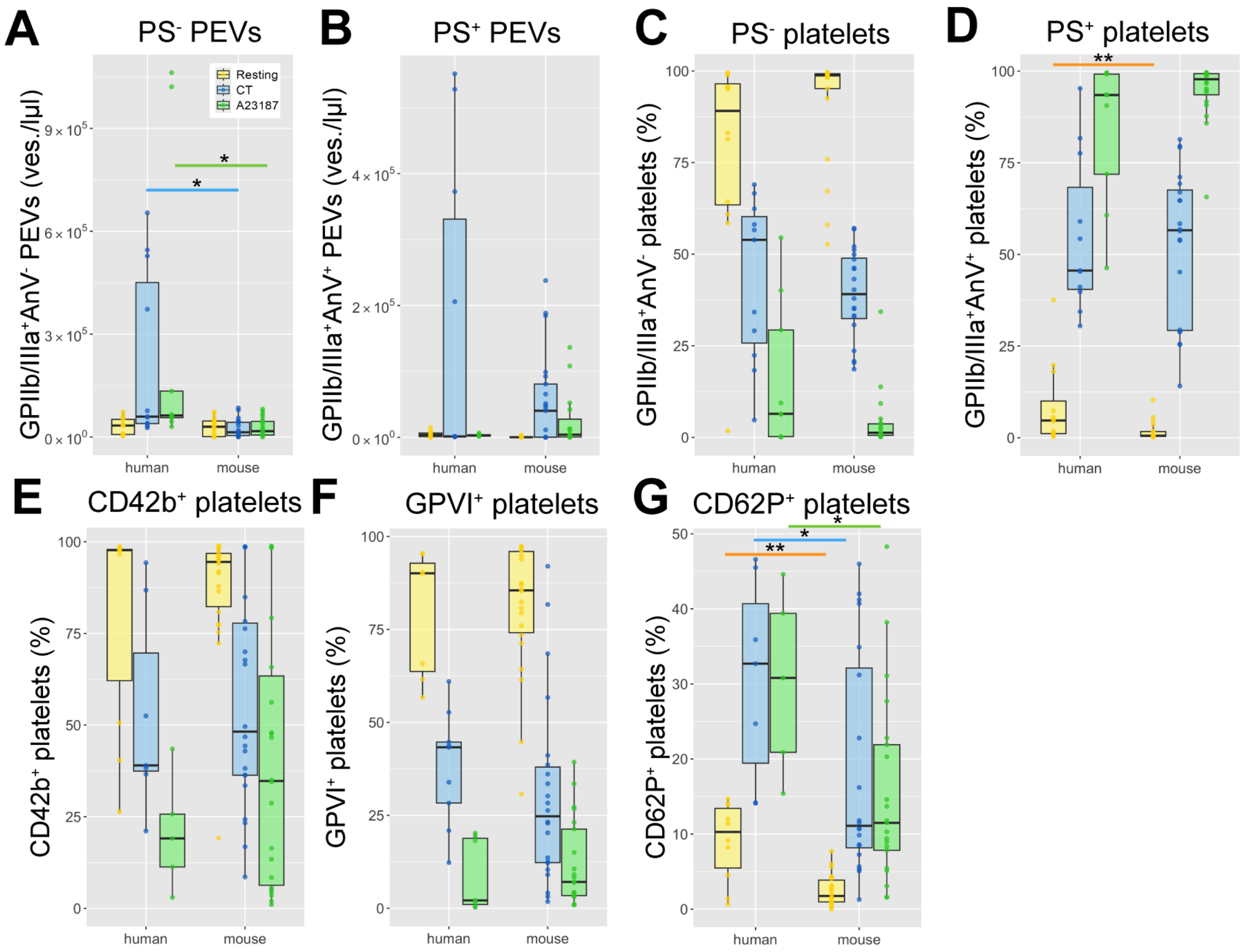
A
PS- PEVs
Resting
CT
A23187
GPIIb/IIIa+AnV- PEVs (ves./µl)
human
mouse
B
PS+ PEVs
GPIIb/IIIa+AnV+ PEVs (ves./µl)
C
PS- platelets
GPIIb/IIIa+AnV- platelets (%)
D
PS+ platelets
GPIIb/IIIa+AnV+ platelets (%)
E
CD42b+ platelets
CD42b+ platelets (%)
F
GPVI+ platelets
GPVI+ platelets (%)
G
CD62P+ platelets
CD62P+ platelets (%)

Figure S4

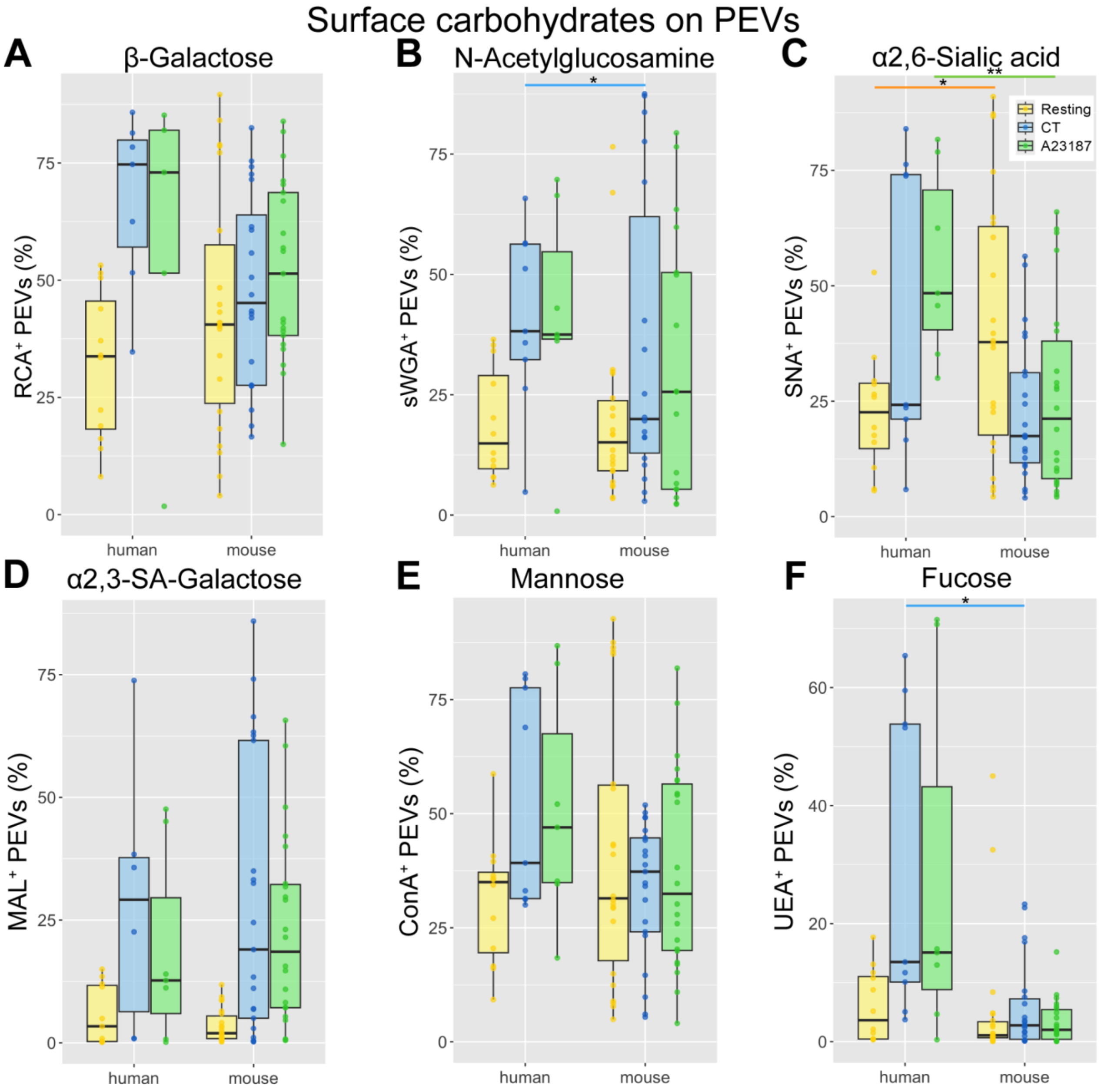
Surface carbohydrates on PEVs
A
β-Galactose
RCA⁺ PEVs (%)
B
N-Acetylglucosamine
sWGA⁺ PEVs (%)
C
α2,6-Sialic acid
SNA⁺ PEVs (%)
Resting
CT
A23187
D
α2,3-SA-Galactose
MAL⁺ PEVs (%)
E
Mannose
ConA⁺ PEVs (%)
F
Fucose
UEA⁺ PEVs (%)
human
mouse

Figure S5

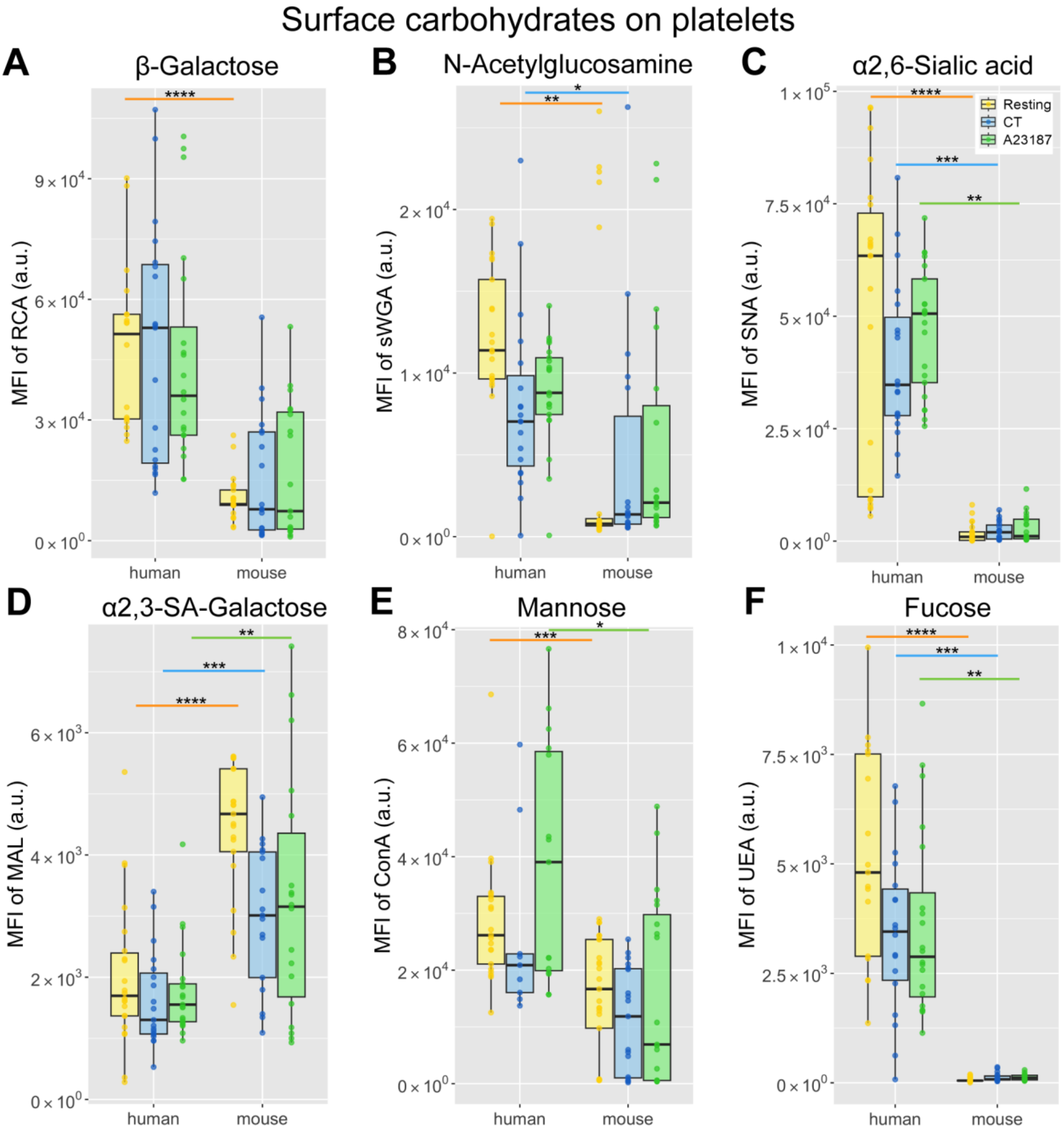

Surface carbohydrates on platelets
A
β-Galactose
MFI of RCA (a.u.)
B
N-Acetylglucosamine
MFI of sWGA (a.u.)
C
α2,6-Sialic acid
MFI of SNA (a.u.)
Resting
CT
A23187
D
α2,3-SA-Galactose
MFI of MAL (a.u.)
E
Mannose
MFI of ConA (a.u.)
F
Fucose
MFI of UEA (a.u.)
human
mouse

Figure S6

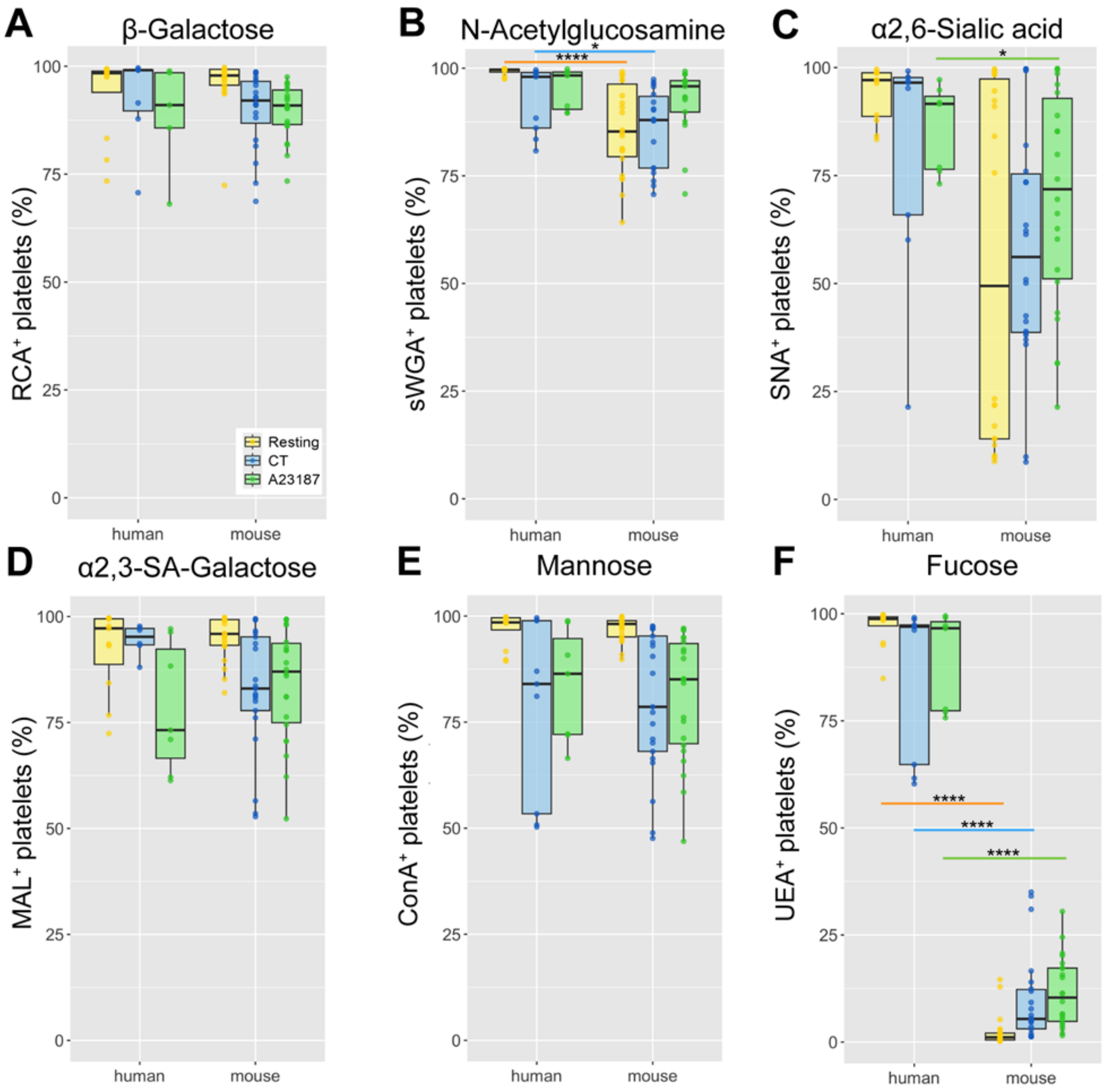

A
β-Galactose
RCA⁺ platelets (%)
100
75
50
25
0
human
mouse
Resting
CT
A23187
B
N-Acetylglucosamine
sWGA⁺ platelets (%)
*
****
C
α2,6-Sialic acid
SNA⁺ platelets (%)
*
D
α2,3-SA-Galactose
MAL⁺ platelets (%)
E
Mannose
ConA⁺ platelets (%)
F
Fucose
UEA⁺ platelets (%)
****
****
****

Figure S7

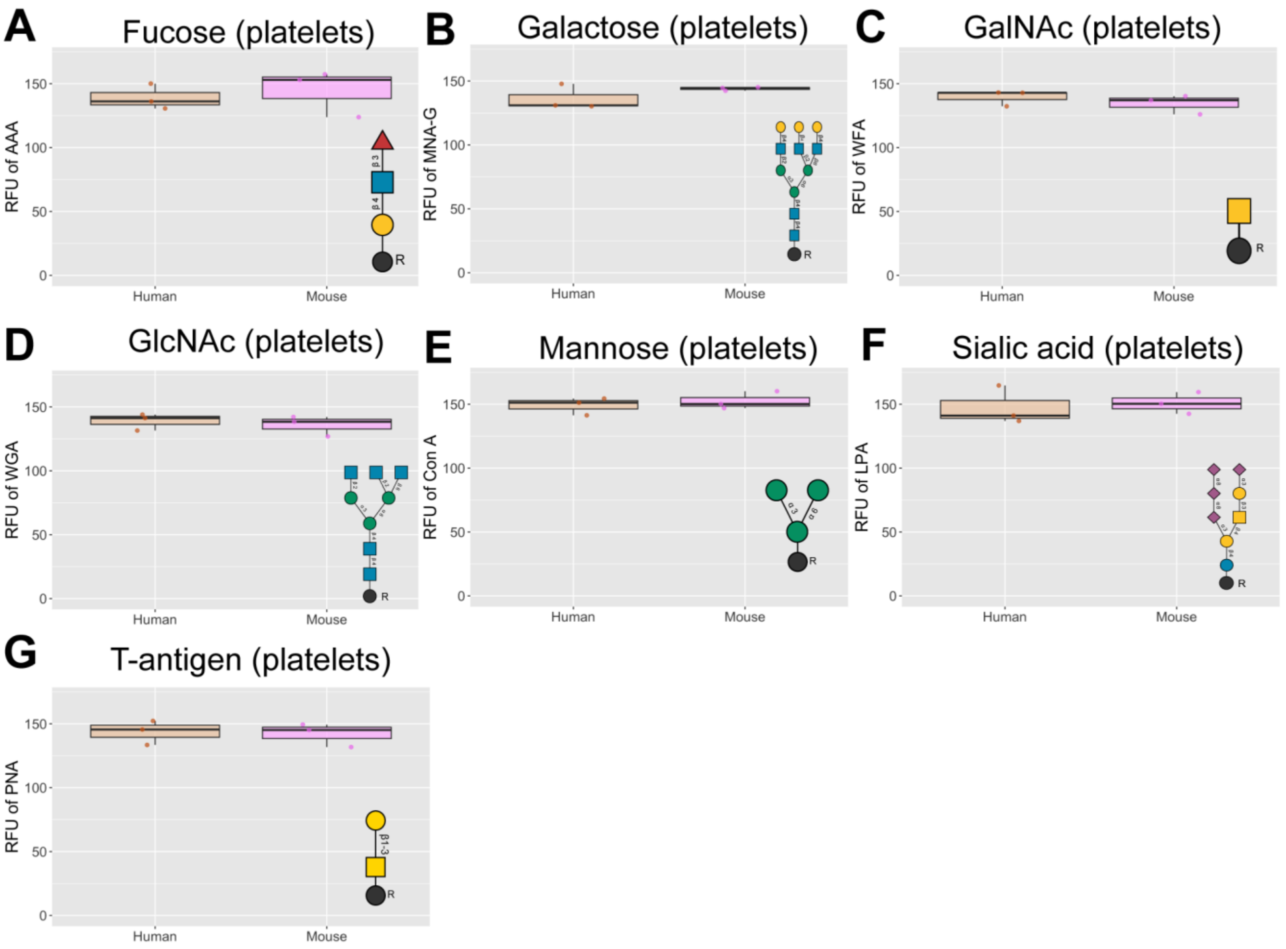
A
Fucose (platelets)
RFU of AAA
150
100
50
0
Human
Mouse
B
Galactose (platelets)
RFU of MNA-G
C
GalNAc (platelets)
RFU of WFA
D
GlcNAc (platelets)
RFU of WGA
E
Mannose (platelets)
RFU of Con A
F
Sialic acid (platelets)
RFU of LPA
G
T-antigen (platelets)
RFU of PNA
R

Figure S8

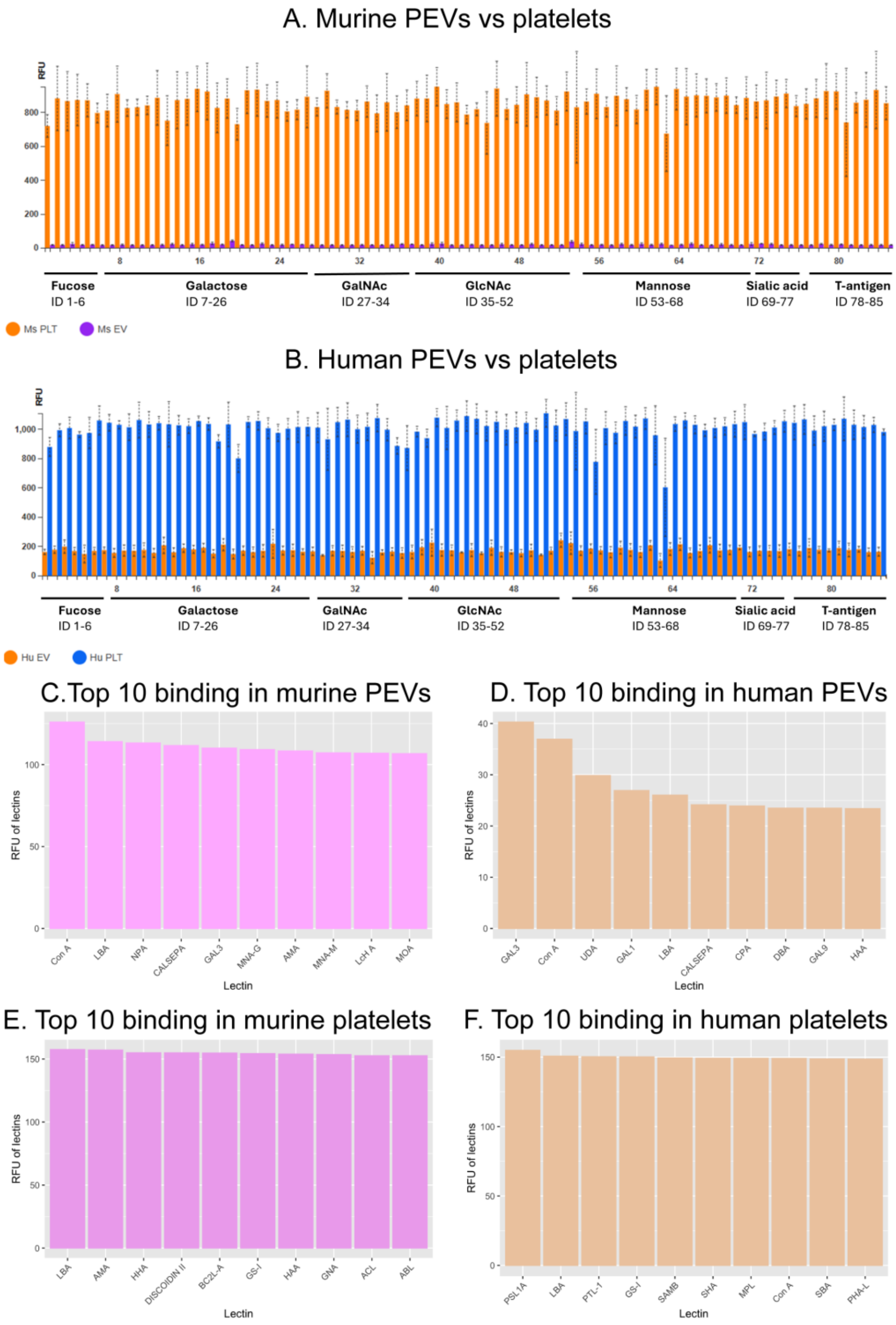
A. Murine PEVs vs platelets
RFU
800
600
400
200
0
8
16
24
32
40
48
56
64
72
80
Fucose
ID 1-6
Galactose
ID 7-26
GalNAc
ID 27-34
GlcNAc
ID 35-52
Mannose
ID 53-68
Sialic acid
ID 69-77
T-antigen
ID 78-85
Ms PLT
Ms EV
B. Human PEVs vs platelets
RFU
1,000
800
600
400
200
0
8
16
24
32
40
48
56
64
72
80
Fucose
ID 1-6
Galactose
ID 7-26
GalNAc
ID 27-34
GlcNAc
ID 35-52
Mannose
ID 53-68
Sialic acid
ID 69-77
T-antigen
ID 78-85
Hu EV
Hu PLT
C.Top 10 binding in murine PEVs
RFU of lectins
100
50
0
Con A
LBA
NPA
CALSEPA
GAL3
MNA-G
AMA
MNA-M
LcH A
MOA
Lectin
D. Top 10 binding in human PEVs
RFU of lectins
40
30
20
10
0
GAL3
Con A
UDA
GAL1
LBA
CALSEPA
CPA
DBA
GAL9
HAA
Lectin
E. Top 10 binding in murine platelets
RFU of lectins
150
100
50
0
LBA
AMA
HHA
DISCOIDIN II
BC2L-A
GS-I
HAA
GNA
ACL
ABL
Lectin
F. Top 10 binding in human platelets
RFU of lectins
150
100
50
0
PSL1A
LBA
PTL-1
GS-I
SAMB
SHA
MPL
Con A
SBA
PHA-L
Lectin

Figure S9

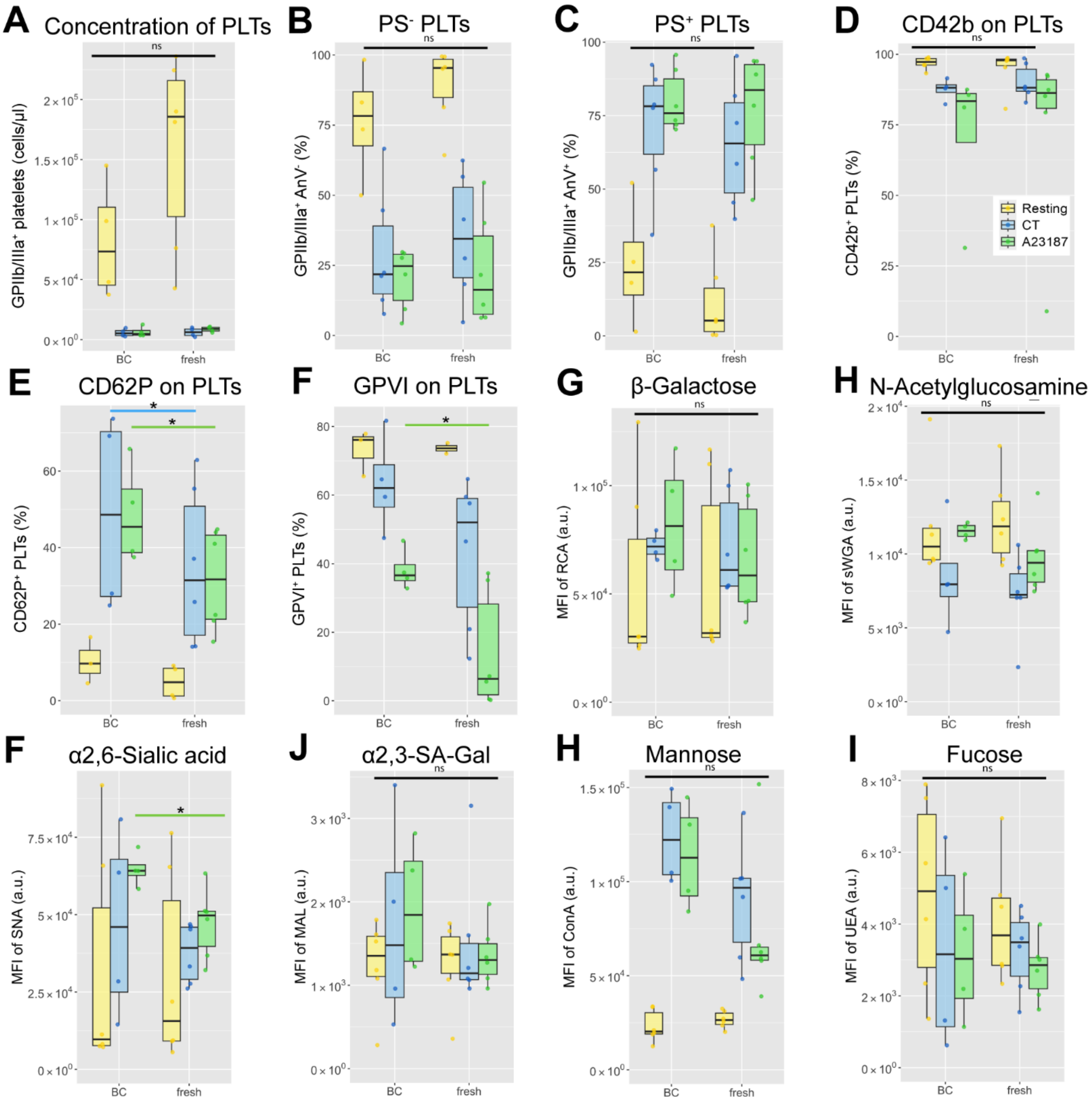

A
Concentration of PLTs
GPIIb/IIIa⁺ platelets (cells/µl)
B
PS⁻ PLTs
GPIIb/IIIa⁺ AnV⁻ (%)
C
PS⁺ PLTs
GPIIb/IIIa⁺ AnV⁺ (%)
D
CD42b on PLTs
CD42b⁺ PLTs (%)
Resting
CT
A23187
E
CD62P on PLTs
CD62P⁺ PLTs (%)
F
GPVI on PLTs
GPVI⁺ PLTs (%)
G
β-Galactose
MFI of RCA (a.u.)
H
N-Acetylglucosamine
MFI of sWGA (a.u.)
F
α2,6-Sialic acid
MFI of SNA (a.u.)
J
α2,3-SA-Gal
MFI of MAL (a.u.)
H
Mannose
MFI of ConA (a.u.)
I
Fucose
MFI of UEA (a.u.)
ns
*
BC
fresh

Figure S10

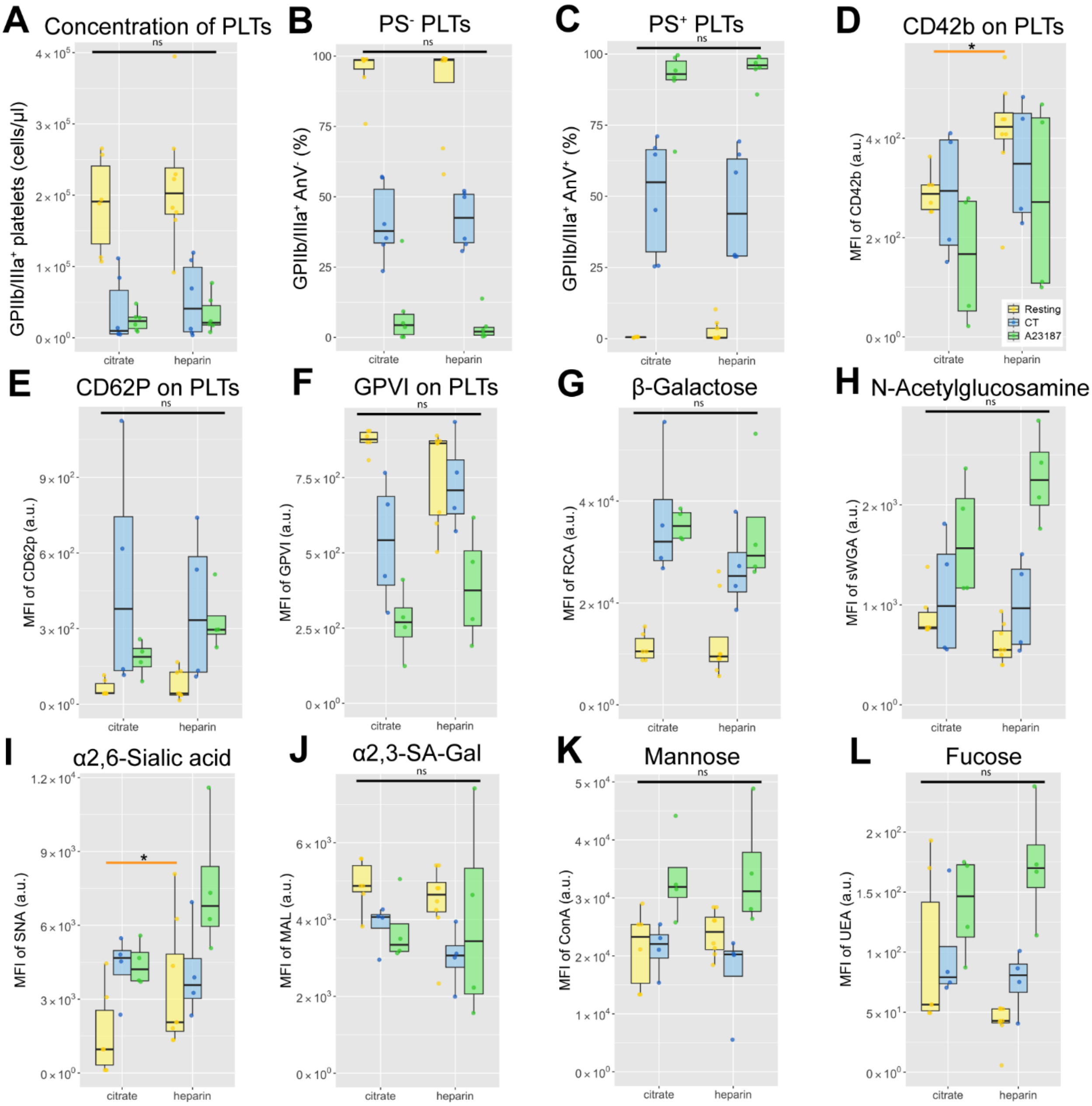
A Concentration of PLTs
GPIIb/IIIa⁺ platelets (cells/µl)
B PS⁻ PLTs
GPIIb/IIIa⁺ AnV⁻ (%)
C PS⁺ PLTs
GPIIb/IIIa⁺ AnV⁺ (%)
D CD42b on PLTs
MFI of CD42b (a.u.)
Resting
CT
A23187
E CD62P on PLTs
MFI of CD62p (a.u.)
F GPVI on PLTs
MFI of GPVI (a.u.)
G β-Galactose
MFI of RCA (a.u.)
H N-Acetylglucosamine
MFI of sWGA (a.u.)
I α2,6-Sialic acid
MFI of SNA (a.u.)
J α2,3-SA-Gal
MFI of MAL (a.u.)
K Mannose
MFI of ConA (a.u.)
L Fucose
MFI of UEA (a.u.)
citrate
heparin
ns
*

MIFlowCyt Checklist

Requirement	Please Include Requested Information
1.1. Purpose	To characterize l-PEVs generated from human and murine platelets using double stimulation of GPVI and PAR1/4 pathways as well as ionophore A23187.
1.2. Keywords	Platelets, extracellular vesicles, clearance of extracellular vesicles, glycans, models of disease
1.3. Experiment variables	Platelet extracellular vesicles (PEVs) were generated from isolated platelets from mouse or human blood. Mouse whole blood was collected by i.v. bleeding into sodium heparin or sodium citrate-containing tubes; human blood was obtained from buffy coats (from healthy donors) stored for 1 day or freshly drawn into 3.8% sodium citrate-containing vacuum tubes. Platelets were isolated using sequential centrifugations and further stimulated for 1 hour at 37°C with either combination of agonists of GPVI- and PAR-receptors or calcium ionophore A23187. Resulting suspension was analyzed using FACSCanto II.
1.4. Organization name and address	Center for Thrombosis and Hemostasis, Medical University of Mainz, Mainz, Germany<br>Address: Langenbeckstrasse 1, 55131, Mainz, Germany
1.5. Primary contact name and email address	Jun.-Prof. Dr. Carsten Deppermann, deppermann@uni-mainz.de
1.6. Date or time period of experiment	2023-2025
1.7. Conclusions	The in-depth comparison of murine and human PEVs revealed major differences of carbohydrate phenotype which provides insight into further biodistribution analysis of platelet-derived extracellular vesicles.
1.8. Quality control measures	The instrument was calibrated using standard calibration beads (BD® CS&T beads). SSC detector was additionally calibrated with Rosetta beads (Exometry, Amsterdam, The Netherlands) to estimate the diameter of measured particles.
2.1.1.1. (2.1.2.1., 2.1.3.1.) Sample description	Samples were isolated platelets containing PEVs after sequential centrifugations, suspensions containing mixtures of PEVs and platelets after 1 hour of stimulation of platelets (see 2.3.). Separation of platelets and PEVs was not performed to avoid interfering with native phenotype of generated particles.
2.1.1.2. Biological sample source description	Mouse whole blood was collected by i.v. bleeding of 10-12 weeks old C57BL/6 mice into sodium heparin or sodium

	citrate-containing tubes; human blood was obtained from buffy coats (from healthy donors) stored for 1 day or peripheral blood from healthy donors was drawn fresh into 3.8% sodium citrate-containing vacuum tubes.
2.1.1.3. Biological sample source organism description	C57BL/6 mice were used to obtain murine samples; healthy human donors were used to obtain human samples.
2.1.2.2. Environmental sample location	NA
2.3. Sample treatment description	$200*10^6$ platelets/mL were stimulated either with 200 µM of PAR1 or 4-AP and 10 µg/mL of collagen-related peptide (CRP-A) or with 20 µM of calcium ionophore A23187 for 1 hour at 37°C upon shaking at 1400 rpm. 5 µl of resulting suspension was added to 95 µl of Annexin Binding buffer in Trucount tubes for analysis of concentration of PEVs and the rest of the suspension was diluted 10 times with Tyrode's buffer containing 2 mM of $CaCl_2$. 45 µl from diluted suspensions were used for each staining with fluorescently-labelled antibodies and lectins. After 20 minutes of staining, samples were diluted 10 times with Tyrode's buffer containing 2 mM of $CaCl_2$ and analyzed at the flow cytometer.<br>Samples were singularly incubated with 5 µg/ml of fluorescein-conjugated lectins (Ricinus communis agglutinin 1 (RCA-1), succinylated Wheat Germ agglutinin (sWGA), Sambucus nigra (SNA), Maackia amurensis (MAL), Concanavalin A (ConA) and Ulex europaeus agglutinin (UAE)). Murine samples were stained with anti-mouse CD42b-DyLight649 (clone Xia.G5, Emfret), GPVI-FITC (clone JAQ1, Emfret), CD62P-FITC (clone Wug.E9, Emfret). Human samples were stained with anti-human CD42b-PE (clone HIP1, BioLegend), Gp6-eFluor660 (clone HY101, Invitrogen), CD62P-AlexaFluor647 (clone AK4, BioLegend).
2.4. Fluorescence reagent(s) description	Lectins are glycoproteins that bind to various structures of carbohydrates. We used them to analyze the surface of l-PEVs to detect the structures known to contribute to platelet clearance. Antibodies against surface platelet antigens were also used to analyze the expression of main platelet glycoproteins on l-PEVs.
3.1. Instrument manufacturer	Becton Dickinson
3.2. Instrument model	FACSCanto II

3.3. Instrument configuration and settings	The instrument is equipped with 488, 633 and 405 nm lasers with 670LP, 585/42, 780/60, 530/30; 780/60, 660/20; 510/50 and 450/50 filters. Side scatter is detected with blue laser through 488/10 filter. Threshold was set to minimal (SSC 200), power of detectors was fixed for all experiments.
4.1. List-mode data files	Data is available under a reasonable request from the corresponding author.
4.2. Compensation description	Compensation was not necessary.
4.3. Data transformation details	Obtained data was calibrated using Mie theory for deriving the estimated diameter in nm. Rosetta beads were recorded for that purpose and Rosetta calibration software (Exometry) was used to add calibration data to the files.
4.4.1. Gate description	Figure S1 provides detailed description of gating strategy.
4.4.2. Gate statistics	Frequency of parent gate and mean fluorescence intensity of used channels were derived. Count of particles was derived from measurement in Trucount tubes for calculation of conentrations.
4.4.3. Gate boundaries	Figure S1 provides detailed description of gating strategy. Lowest estimated diameter limit was 300 nm.

MIFlowCyt-EV Checklist

Framework Criteria	What to report	Please complete each criterion
1.1 Preanalytical variables conforming to MISEV guidelines.	Preanalytical variables relating to EV sample including source, collection, isolation, storage, and any others relevant and available in the performed study.	Platelet-derived extracellular vesicles (PEVs) were not separated form source cells (platelets) for flow cytometry to avoid alternating the surface phenotype. Human samples were derived from buffy coats that were stored for 1day prior usage and platelets were further isolated in presence of 3.8% of sodium citrate. On the other hand, murine samples were obtained from freshly drawn peripheral blood into sodium heparin-containing tubes. Comparison of platelets and l-PEVs from buffy coats and freshly derived human blood and comparison of different anticoagulants (heparin and citrate) did not reveal major differences (Fig. 5, 6).
1.2 Experimental design according to MIFlowCyt guidelines.	EV-FC manuscripts should provide a brief description of the experimental aim, keywords, and variables for the performed FC experiment(s) using MIFlowCyt checklist criteria: 1.1, 1.2, and 1.3, respectively. Template found at www.evflowcytometry.org.	Aim was to characterize l-PEVs generated from human and murine platelets using double stimulation of GPVI and PAR1/4 pathways as well as ionophore A23187
2.1 Sample staining details	State any steps relating to the staining of samples. Along with the method used for staining, provide relevant reagent descriptions as listed in MIFlowCyt guidelines (Section 2.4 Fluorescence Reagent(s) Descriptions).	Number of l-PEVs in the samples was calculated using Trucount tubes (BD, New Jersey, USA). 5 µl of undiluted suspensions of platelets and l-PEVs after 1 hour of generation of l-PEVs was added to 95 µL of Annexin Binding buffer (BioLegend, San Diego, USA), stained with CD41-AlexaFluor647 (anti-mouse) or CD61-FITC (anti-human, clone MwReg30, BioLegend) and AnnexinV-AlexaFluor647 (BioLegend) for 20 minutes at RT. The reaction was stopped with 900µL of Annexin Binding buffer. Samples were singularly incubated with 5 µg/ml of fluorescein-conjugated lectins

		(Ricinus communis agglutinin 1 (RCA-1), succinylated Wheat Germ agglutinin (sWGA), Sambucus nigra (SNA), Maackia amurensis (MAL), Concanavalin A (ConA) and Ulex europaeus agglutinin (UAE)). Murine samples were stained with anti-mouse CD42b-DyLight649 (clone Xia.G5, Emfret), GPVI-FITC (clone JAQ1, Emfret), CD62P-FITC (clone Wug.E9, Emfret). Human samples were stained with anti-human CD42b-PE (clone HIP1, BioLegend), Gp6-eFluor660 (clone HY101, Invitrogen), CD62P-AlexaFluor647 (clone AK4, BioLegend).
2.2 Sample washing details	State any steps relating to the washing of samples.	NA
2.3 Sample dilution details	All methods and steps relating to sample dilution.	Samples were processed using Tyorde's buffer ((140 mM NaCl, 0.5 mM $NaHCO_3$, 3 mM KCl, 0.5 mM $MgCl_2$, 10 mM D-glucose, 10 mM HEPES, pH 7.35) with 2 mM of $CaCl_2$
3.1 Buffer alone controls.	State whether a buffer-only control was analyzed at the same settings and during the same experiment as the samples of interest. If utilized it is recommended that all samples be recorded for a consistent set period of time e.g. 5 minutes, rather than stopping analysis at a set recorded event count e.g. 100,000 events. This allows comparisons of total particle counts between controls and samples.	Buffer-only control was used each experimental day at the same settings, and all events were recorded for 1 minute.
3.2 Buffer with reagent controls.	State whether a buffer with reagent control was analyzed at the same settings, same concentrations, and during the same experiment as the samples of interest. If used state what the results were.	Buffer with staining reagents was analyzed once when the setup of the experiments was performed.

3.3 Unstained controls.	State whether unstained control samples were analyzed at the same settings and during the same experiment as stained samples. If used, state what the results were, preferably in standard units.	Unstained control was recorded every experimental day and 10,000 events in EV gate were recorded.
3.4 Isotype controls.	The use of isotype controls is applicable to immunofluorescence labelling only. State whether isotype controls were analyzed at the same settings and during the same experiment as stained samples. If utilized, state which antibody they are matched to, the concentration used, and what the results were (Section 4.2, 4.3, 4.4). Due to conjugation differences between manufacturers if should be stated if the isotype controls are from the same manufacturer as the matched antibodies.	Respective isotype controls for each antibody were used every experimental day and 10,000 events in EV gate were recorded.
3.5 Single-stained controls.	State whether single-stained controls were included. If used state whether the single-stained controls were recorded using the same settings, dilutions, and during the same experiment as stained samples and state what the results were, preferably in standard units (Section 4.2, 4.3, 4.4).	Majority of samples were analyzed using single stainings. In Trucount tubes particles were stained with CD61-FITC and AnnexinV-AlexaFluor647 (human samples) or CD41-AlexaFluor647 and AnnexinV-FITC (mouse samples).
3.6 Procedural controls.	State whether procedural controls were included. If used, state the procedure and if the procedural controls were acquired at the same settings and during the same experiment as stained samples.	Shaking of resting platelets in absence of activators was performed to control the effect of shaking on platelet vesiculation.
3.7 Serial dilutions.	State whether serial dilutions were performed on samples and note the dilution range and manner of testing. The fluorescence and/or scatter signal intensity would ideally be reported in standard units (see Section 4.3, 4.4) but arbitrary units can also be used. This data is best reported by plotting the recorded number events/concentration	Serial dilutions were not performed.

	over a set period of time at different sample dilution. The median fluorescence intensity at each of the dilutions should also ideally be plotted on the same or a separate plot.	
3.8. Detergent treated EV-samples	State whether samples were detergent treated to assess lability. If utilized, state what detergent was used, the end concentration of the detergent, and what the results were of the lysis.	Samples were treated with 1% of Triton-X100 (Fig. S1). Incubation was performed for 60 minutes at room temperature and resulted in significant decrease in amount of observed events.
4.1 Trigger Channel(s) and Threshold(s).	The trigger channel(s) and threshold(s) used for event detection. Preferably, the fluorescence calibration (Section 4.3) and/or scatter calibration (Section 4.4) should be used in order to report the trigger channel(s) and threshold(s) in standardized units.	Side scatter from red laser was used as a trigger channel. Rosetta calibration was used to estimate the diamemter of observed events. Threshold was set to the lowest possible (SSC 200).
4.2 Flow Rate / Volumetric quantification.	State if the flow rate was quantified/validated and if so, report the result and how they were obtained.	Measurements were performed at low (10 µl/min) or medium (60 µl/min) flow rate with target speed range of 200-900 events/s. Flow rate was controlled by instrument.
4.3 Fluorescence Calibration.	State whether fluorescence calibration was implemented, and if so, report the materials and methods used, catalogue numbers, lot numbers, and supplied reference units for the standards. Fluorescence parameters may be reported in standardized units of MESF, ERF, or ABC beads. The type of regression used, and the resulting scatter plot of arbitrary data vs standard data for the reference particles should be supplied.	Fluorescence calibration was not performed.
4.4 Light Scatter Calibration.	State whether and how light scatter calibration was implemented. Light scatter parameters may be reported in standardized units of nm2, along with	Light scatter was calibrated with Rosetta calibration beads. The beads were recorded at experimental settings. Rosetta calibration software was used to apply Mie theory to the experimental samples to

	information required to reproduce the model.	use the estimated diameter for gating the EVs (<1um).
5.1 EV diameter/surface area/volume approximation.	State whether and how EV diameter, surface area, and/or volume has been calculated using FC measurements.	Rosetta calibration was used for estimation of EV diameter.
5.2 EV refractive index approximation.	State whether the EV refractive index has been approximated and how this was done.	EV refractive index approximation was not performed.
5.3 EV epitope number approximation.	State whether EV epitope number has been approximated, and if so, how it was approximated.	EV epitope numbers approximation was not performed.
6.1 Completion of MIFlowCyt checklist.	Complete MIFlowCyt checklist criteria 1 to 4 using the MIFlowCyt guidelines. Template found at www.evflowcytometry.org.	MIFlowCyt checklist has been completed.
6.2 Calibrated channel detection range	If fluorescence or scatter calibration has been carried out, authors should state whether the upper and lower limits of a calibrated detection channel were calculated in standardized units. This can be done by converting the arbitrary unit scale to a calibrated scaled, as discussed in Section 4.3 and 4.4, and providing the highest unit on this scale and the lowest detectable unit above the unstained population. The lowest unit at which a population is deemed 'positive' can be determined a variety of ways, including reporting the 99th percentile measurement unit of the unstained population for fluorescence. The chosen method for determining at what unit an event was deemed positive should be clearly outlined.	Fluorescence limits were not assessed. Lowest limit of diameter detected was estimated 300 nm.
6.3 EV number/concentration.	State whether EV number/concentration has been reported. If calculated, it is preferable to report EV number/concentration in a standardized	BD Trucount tubes were used to calculate the concentration of generated l-PEVs. Sysmex KXN-1 hematologic analyzer

	manner, stating the number/concentration between a set detection range.	was used after platelet isolation to determine the platelet concentration.
6.4 EV brightness.	When applicable, state the method by which the brightness of EVs is reported in standardized units of scatter and/or fluorescence.	NA
7.1. Sharing of data to a public repository.	Provide a link to the experimental data in a public data repository.	Data is available under a reasonable request from the corresponding author.
